# Improving the Dung Beetle Optimizer with Multiple Strategies: An Application to Complex Engineering Problems

**DOI:** 10.3390/biomimetics10110717

**Published:** 2025-10-23

**Authors:** Wei Lv, Yueshun He, Yuankun Yang, Xiaohui Ma, Jie Chen, Yuxuan Zhang

**Affiliations:** 1 School of Artificial Intelligence and Information Engineering, East China University of Technology, Nanchang 330013, China; 2023110202@ecut.edu.cn (W.L.); 2023110197@ecut.edu.cn (Y.Y.); 2023110187@ecut.edu.cn (X.M.); asd444324202@126.com (Y.Z.); 2 School of Surveying and Geoinformation Engineering, East China University of Technology, Nanchang 330013, China; codercjie@ecut.edu.cn

**Keywords:** DBO, oscillating balance factor, improved dung beetle foraging strategy, multi-population differential co-evolutionary mechanism, engineering optimization problems

## Abstract

Although the Dung Beetle Optimizer (DBO) is a promising new metaheuristic for global optimization, it often struggles with premature convergence and lacks the necessary precision when applied to complex optimization challenges. Therefore, we developed the Multi-Strategy Improved Dung Beetle Optimizer (MIDBO), an algorithm that incorporates several new strategies to enhance the performance of the standard DBO. The algorithm enhances initial population diversity by improving the distribution uniformity of the Circle chaotic map and combining it with a dynamic opposition-based learning strategy for initialization. A nonlinear oscillating balance factor and an improved foraging strategy are introduced to achieve a dynamic equilibrium between the algorithm’s global search and local refinement, thereby accelerating convergence. A multi-population differential co-evolutionary mechanism is designed, wherein the population is partitioned into three categories according to fitness, with each category using a unique mutation operator to execute targeted searches and avoid local optima. A comparative study against multiple metaheuristics on the CEC2017 and CEC2022 benchmarks was performed to comprehensively evaluate MIDBO’s performance. The practical effectiveness of the MIDBO algorithm was validated by applying it to three practical engineering challenges. The results demonstrate that MIDBO significantly outperformed the other algorithms, a success attributed to its superior optimization performance.

## 1. Introduction

An optimization algorithm seeks to identify the set of parameters that produces the optimal value (maximum or minimum) for an objective function, subject to a series of defined constraints [1]. Conventional optimization algorithms are typically contingent upon specific mathematical models or problem structures, which enable them to efficiently find the optimal solution for low-dimensional, well-structured, or gradient-based problems like linear, convex, and continuous optimization [2,3]. Nevertheless, optimization problems in practical applications exhibit significant diversity and are classified into multiple types based on whether they are single-objective or multi-objective, static or dynamic, and discrete or continuous [4]. The hallmarks of these problems include non-convexity, nonlinear constraints, high dimensionality, and substantial computational expense, with the difficulty of finding a solution escalating as the problem’s dimensions grow [5]. Therefore, employing traditional algorithms for such challenges is often problematic due to high computational complexity, the risk of stagnation at suboptimal solutions, and an inability to achieve the level of precision demanded by real-world engineering problems [6].

Against this backdrop, metaheuristic algorithms have seen widespread adoption in various optimization problems, attributable to their ease of implementation, their non-reliance on gradient data, and their lower susceptibility to premature convergence [7]. Metaheuristic algorithms are distinguished from traditional methods by their use of a search mechanism based on predefined rules and stochastic operators that, by operating independently of the search space’s gradient information, enables them to solve most real-world non-convex, nonlinear, and high-dimensional optimization problems [3]. Metaheuristic algorithms can find a solution at or near the global optimum under more complex constraints. Therefore, this class of algorithms is better suited for solving optimization tasks with complex constraint conditions.

Based on the description above, ongoing progress in metaheuristic optimization has spurred the emergence of various pioneering algorithms, which can be grouped into four classes based on their characteristics. The first class of algorithms is derived from principles of biological evolution, including Biogeography-based optimization (BBO) [8] and differential evolution (DE) [9]. A second category of algorithms draws from physical laws or mathematical principles, featuring algorithms such as the sine cosine algorithm (SCA) [10], thermal exchange optimization (TEO) [11], atom search optimization (ASO) [12], and Keplerian optimization algorithm (KOA) [13]. Human social behaviors provide the conceptual basis for the third category, which includes social group optimization (SGO) [14], league championship algorithm (LCA) [15], and the student psychology optimization (SPO) algorithm [16]. Lastly, swarm intelligence-based methods constitute the fourth class, including beluga whale optimization (BWO) [17], the walrus optimizer (WO) [18], the hippopotamus optimization algorithm (HO) [19], and Greylag Goose Optimization (GGO) [20].

While the multitude of optimization algorithms documented in prior research each possess distinct merits, according to the no free lunch (NFL) theorem, there is no one-size-fits-all algorithm that is universally optimal for every kind of optimization problem [21]. This theorem justifies the creation of domain-specific solutions, since an algorithm’s effectiveness on a particular set of problems does not guarantee similar success in other areas [22]. Consequently, motivated by the NFL theorem, researchers have spent the last few decades developing numerous algorithmic variants designed to advance the efficacy of metaheuristic approaches.

As an illustration, Xie et al. [23] developed a sparrow search algorithm enhanced with a dynamic classification mechanism. The specific improvements include (1) employing an elite opposition-based Chebyshev strategy when generating the initial population to ensure the starting solutions are both diverse and well-distributed, thereby preventing premature convergence, and (2) proposing a dynamic multi-subpopulation classification mechanism that stratifies the sparrow population into elite, intermediate, and inferior subgroups, with stratification dictated by individual fitness values. The elite subgroup employs an elite guidance strategy to accelerate the convergence process; the intermediate subgroup applies an adaptive dynamic inertia weight strategy, guiding it in balancing its exploratory and exploitative behaviors; and the inferior subgroup aims to enhance final convergence precision by employing a golden sine strategy. Zhu et al. [24] modified the snake optimizer with the addition of several new strategies. Several specific improvements were made, which are detailed below and include the following: (1) enhancing the quality of the algorithm’s initial solution by designing a multi-seed chaotic mapping mechanism to generate the initial population, resulting in a more homogenous dispersal of the starting individuals, (2) incorporating an anti-predation strategy within the exploration process, aiming to widen the searchable area while enhancing the rate and precision of convergence, and (3) proposing a bidirectional population evolution dynamics strategy aimed at bolstering local exploitation process, thereby steering the algorithm away from premature convergence and improving the equilibrium between its global and local search capabilities. Similarly, an adaptive and diversified version of the hiking optimization algorithm was introduced by Abdel-salam et al. [25]. Their specific improvements include (1) proposing a stratified random initialization strategy to be employed during initial population construction, thereby promoting greater diversity within the set of starting solutions, (2) proposing an enhanced leader coordination strategy to mitigate the risk of premature convergence, (3) an adaptive perturbation strategy introduced as a solution to local optima entrapment, aiming to improve the hiking optimization algorithm’s escape capability, and (4) employing a dynamic exploration strategy to maintain a good trade-off between the algorithm’s exploratory and exploitative phases.

A recent addition to the swarm intelligence family, the Dung Beetle Optimizer (DBO) [26], is an algorithm which draws its core principles from the life habits of dung beetles. It establishes an innovative search framework by simulating their behaviors of ball-rolling, dancing, breeding, foraging, and stealing. Distinguishing itself from other prominent swarm intelligence techniques, the DBO features a unique search process that adeptly manages the trade-off between global search and local refinement, resulting in both rapid convergence and high-precision solutions [27]. As a result, researchers have applied the DBO to solve numerous practical problems.

For instance, the authors of [28] applied an elite-based variant of the DBO to fine-tune the hyperparameters of an encrypted traffic classification model based on a multi-scale convolutional neural network. For the purpose of boosting the quantity and rated power of fuel cell stacks, the work in [29] utilized the DBO for the optimization of a manifold’s structural parameters, thereby improving the consistency of the fuel cell’s performance. In [30], a composite model combining a self-attention temporal convolutional network and a bidirectional long short-term memory network was developed for ultra-short-term photovoltaic power forecasting; this model’s hyperparameters were subsequently fine-tuned using the DBO for improved predictive accuracy. Furthermore, [31] developed an improved variant of the DBO specifically for application in search and rescue operations that utilize multiple UAVs within disaster environments, and the authors succeeded in finding the most efficient route in minimal time. Lastly, [32] optimized a long short-term memory model using the DBO which, when used in conjunction with the variational mode decomposition technique, predicted methane production from deep coal seams and improved the precision of daily yield forecasts.

Despite its superior performance relative to many algorithms in the swarm intelligence family in tackling specific optimization challenges, it also possesses certain shortcomings. The authors of [33] pointed out three drawbacks of the DBO. (1) The canonical DBO algorithm begins by creating its initial population through a random initialization method. The diversity of the initial population may be compromised because this stochastic approach can result in poor spatial coverage of individuals throughout the search space. (2) The foraging dung beetles’ lack of adaptability impairs their global exploration capability and increases their propensity for premature convergence. (3) Lastly, the strategy used to adjust the locations of thieving dung beetles is reliant on the current individual’s best solution, a factor that diminishes population diversity and reduces the convergence precision. The authors of [34] noted that the reliance of the position update rule for ball-rolling dung beetles in the global worst position impairs the algorithm’s global search performance and undermines the balance between its exploratory and exploitative capabilities. As pointed out in [35], the linear adjustment of parameter R as part of the dung beetle’s reproduction strategy, while straightforward, elevates the likelihood of the algorithm converging prematurely and may prevent the discovery of more optimal solutions. In summary, further enhancements can be made to the DBO to elevate its performance in optimization tasks.

This paper proposes an enhanced variant of the standard DBO with multiple strategies. The goal of this new algorithm is to address key weaknesses such as its population initialization, balancing exploration and exploitation, and its susceptibility to premature convergence. The algorithm enhances population diversity and augments the global search capabilities of the baseline algorithm by incorporating an efficient initialization mechanism and a dynamic balancing strategy. Ultimately, these enhancements enable the algorithm to surmount the challenge of premature convergence, thereby elevating its solution accuracy when tackling complex optimization problems. Through these improvements, this paper makes the following key contributions:

• An enhanced version of the DBO algorithm, named MIDBO, is proposed, which improves overall performance by integrating four new strategies into the original framework.
• We validate the numerical optimization effectiveness of the MIDBO on functions selected from the CEC2017 and CEC2022 benchmark collections and in comparison with six competing algorithms. Based on the experimental data, the MIDBO possesses a clear competitive edge.
• To further test the MIDBO’s practical utility, it was applied to three practical engineering challenges, where its success in securing optimal solutions underscored its high performance when tackling complex challenges.

The subsequent sections of this paper are as follows. Section 2 introduces the standard DBO. Section 3 introduces the new improvement mechanisms in the proposed MIDBO. Section 4 presents comparative experiments involving the MIDBO against other optimization algorithms on two test suites. Section 5 applies the MIDBO to specific engineering application scenarios. Section 6 summarizes the paper and offers an outlook on potential future work.

## 2. DBO

The core inspiration for the dung beetle optimization algorithm stems from several natural activities of this species, namely rolling, dancing, foraging, stealing, and reproduction. From these habits, four distinct population update mechanisms are derived.

### 2.1. Rolling Dung Beetles

The act of rolling a dung ball to an appropriate spot is a natural behavior exhibited by dung beetles. The purpose of acquiring a dung ball is twofold: (1) for laying eggs and raising offspring and (2) as a source of food. To maintain a straight trajectory when rolling a dung ball, a beetle uses celestial cues—like the sun and moon—for orientation. To model this process, the algorithm randomly chooses a direction from the entire search space and maintains a straight path. Equations (1) and (2) describe the position updating in an obstacle-free environment:

(1) $$X_{i}\left(t+1\right)=X_{i}\left(t\right)+\alpha \times k\times X_{i}\left(t-1\right)+b\times \Delta x$$

(2) $$\Delta x=|X_{i}\left(t\right)-X^{W}|$$

In this formulation, the current iteration is denoted by *t*, and the position of the *i*th dung beetle is given by $X_{i}\left(t\right)$. The equation uses a constant deflection coefficient k∈(0,0.2] and a random number *b* from the interval (0, 1). The global worst position and a simulation of light intensity changes are represented by $X^{W}$ and Δx, respectively. A deviation coefficient α is also used, whose value is assigned based on the probabilistic condition in Equation (3):

(3) $$\alpha =\left\{\begin{array}{cc} 1 & \lambda >\eta \\ -1 & \lambda \leq \eta \end{array}\right.$$

In the formula, λ is a random number in the range [0,1], and η is a probability value set to 0.1. A value of 1 signifies that the beetle does not deviate from the forward direction, while −1 denotes a deviation of the beetle’s path from the target.

When a dung beetle’s rolling path is obstructed, it performs a characteristic reorientation behavior, often described as a “dance”, to establish a new direction. Equation (4) describes this dancing behavior:

(4) $$X_{i}\left(t+1\right)=X_{i}\left(t\right)+\tan\left(\theta \right)\left|X_{i}\left(t\right)-X_{i}\left(t-1\right)\right|$$

When the parameter θ equals 0, π/2, or π, the position is not altered; otherwise, θ is constrained within the range [0, π].

### 2.2. Breeding Dung Beetles

Dung beetles in the wild will deliberately choose a protected area for laying their eggs. The source algorithm models this process using a boundary selection strategy, with its formula presented as follows in Equation (5):

(5) $$\left\{\begin{array}{cc} Lb^{*} & =\max(X_{best}^{l}\times \left(1-R\right),Lb)\\ Ub^{*} & =\min(X_{best}^{l}\times \left(1+R\right),Ub) \end{array}\right.$$

wherein the egg-laying area is constrained by the lower and upper bounds $Lb^{*}$ and $Ub^{*}$, while the current local best position is denoted by $X_{best}^{l}$. The equation also uses a dynamic parameter *R*, which decreases over time according to $R=1-\frac{t}{M}$ (where *M* is the maximum number of iterations). Finally, Lb and Ub represent the lower and upper bounds for the entire search space, respectively.

Equation (5) indicates that the entire oviposition region is dynamic and varies as a function of *R*. Equation (6) provides the definition of the spawning location:

(6) $$B_{i}\left(t+1\right)=X_{best}^{l}+m_{1}\times \left(B_{i}\left(t\right)-Lb^{*}\right)+m_{2}\times \left(B_{i}\left(t\right)-Ub^{*}\right)$$

Two independent random vectors, $m_{1}$ and $m_{2}$, are used in the equation. Each vector has a size of 1×D, corresponding to the dimensionality (*D*) of the search space for the optimization problem.

### 2.3. Small Dung Beetles

Newly hatched dung beetles emerge from underground and begin to forage for food. To simulate this natural behavior, an optimal foraging region is delineated to guide their search. This region is mathematically defined by Equation (7):

(7) $$\left\{\begin{array}{cc} Lb^{b} & =\max(X_{best}^{g}\times \left(1-R\right),Lb)\\ Ub^{b} & =\min(X_{best}^{g}\times \left(1+R\right),Ub) \end{array}\right.$$

wherein, the optimal foraging region is bounded by the lower and upper limits $Lb^{b}$ and $Ub^{b}$, respectively, and the global best position is represented by $X_{best}^{g}$. The other parameters are consistent with their definitions in Equation (5). Equation (8) then provides the rule for updating the locations of small dung beetles:

(8) $$X_{i}\left(t+1\right)=X_{i}\left(t\right)+K_{1}\times \left(X_{i}\left(t\right)-Lb^{b}\right)+K_{2}\times \left(X_{i}\left(t\right)-Ub^{b}\right)$$

wherein, $K_{1}$ is a random number following a normal distribution and $K_{2}$ is a random vector within (0,1).

### 2.4. Thieving Dung Beetles

A subset of the population, designated as “thieving dung beetles” (or “thieves”), engages in stealing dung balls. Based on the optimal foraging region defined in Equation (7), this competitive behavior occurs mainly in the area surrounding the global best position ($X_{best}^{g}$). Consequently, the new position for these thieves is determined by Equation (9):

(9) $$X_{i}\left(t+1\right)=X_{best}^{g}+S\times g\times \left(|X_{i}\left(t\right)-X_{best}^{l}\left|+\right|X_{i}\left(t\right)-X_{best}^{g}|\right)$$

wherein, *S* is defined as a constant, while *g* denotes a random vector with a size of 1×D sampled from a normal distribution.

### 2.5. Pseudo-Code of DBO

The pseudo-code of the DBO is shown as Algorithm 1.

**Algorithm 1** The framework of the DBO algorithm **Require:** The maximum iteration $T_{max}$, the size of the particle’s population *N* **Ensure:** Optimal position $X_{best}^{g}$ and its fitness value $f_{min}$     1:   Initialize the particle’s population i←1,2,…,N and define its relevant parameters     2:  
** while** $t\leq T_{max}$
** do**     3:     **for** i=1 to Number of rolling dung beetles **do**     4:        a=rand(1)     5:        **if** a≤0.9 **then**     6:           Update the rolling dung beetle’s position by using Equations (1) and (2)     7:  
      **else**     8:           Update the rolling dung beetle’s position by using Equation (4)     9:        **end if**   10:     **end for**   11:     **for** i=1 to Number of breeding dung beetles **do**   12:        Update breeding dung beetle’s position by using Equations (5) and (6)   13:     **end for**   14:     **for** i=1 to Number of small dung beetles **do**   15:        Update small dung beetle’s position by using Equations (7) and (8)   16:     **end for**   17:     **for** i=1 to Number of stealing Dung Beetles **do**   18:        Update stealing dung beetle’s location by using Equation (9)   19:     **end for**   20:     t=t+1   21:  **end while**   22:  **return **$X_{best}^{g}$ and its fitness value $f_{min}$

## 3. Proposed Algorithm

### 3.1. Population Initialization Based on Chaotic Opposition-Based Learning Strategy

The efficiency of swarm intelligence optimization algorithms is influenced by its population initialization, as a well-distributed initial population improves the coverage of the solution space, which in turn enhances both the convergence speed and precision [36]. The conventional DBO typically employs a uniform random initialization method to form its initial swarm, and while this stochastic nature is beneficial for exploring disparate zones within the search space and improves the prospects for discovering the global optimal solution, it presents a notable shortcoming. Specifically, there is no mechanism to secure a homogenous distribution, which often results in high-density pockets of candidates in some regions and low-density voids in others. Such an imbalanced spread poses a significant impediment to the algorithm’s initial convergence phase. To boost the quality of the initial candidate solutions, this study introduces a hybrid initialization strategy that fuses chaotic maps with dynamic opposition-based learning (OBL).

**Figure 1:**
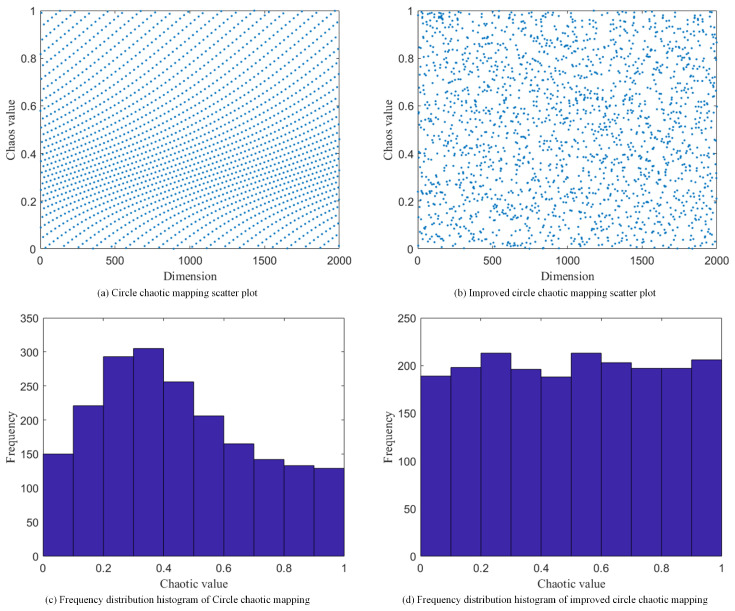
Scatter plot and frequency distribution histogram for the improved circle chaotic map.

Compared with random initialization, chaotic maps exhibit superior performance in optimization searches, especially when locating the global optimum, an advantage that stems from their comprehensive traversal of the search domain and their effectiveness at evading local optima, thereby enabling a more thorough exploration of the solution domain [37]. The circle map, when compared with other prevalent chaotic maps like the Chebyshev, logistic, and tent maps, has gained attention for its strong ergodicity, high coverage of chaotic values, and strong stability [38,39]. However, experiments revealed that the values generated by the circle map tend to cluster heavily within the interval [0.2, 0.5], making its distribution non-uniform. For the purpose of improving the uniformity of its chaotic value distribution, a modified circle map formula was developed. Equation (10) presents the original formulation of the circle chaotic map:

(10) $$x_{n+1}=\mathrm{mod}\left(x_{n}+0.2-\frac{0.5}{2\pi }\sin\left(2\pi x_{n}\right),1\right)$$

wherein, $x_{n}$ represents the nth chaotic sequence number. The scatter plot and frequency histogram corresponding to the standard circle chaotic map are presented in Figure 1a,c, respectively.

As shown in Figure 1a,c, the standard circle chaotic map generates values that are predominantly concentrated within the [0.2, 0.5] interval. Such a dense clustering of initial candidate solutions compromises the DBO’s population diversity. This study addresses this limitation by proposing a modified circle chaotic map that alters the original formula in three primary ways: (1) the linear term $x_{n}$ is scaled by a factor of four; (2) the influence of the sine term is amplified by augmenting its coefficient from 0.5 to 0.8; and (3) the constant terms are reconfigured to 0.3 and 0.25π. The resulting modified map yields superior chaotic properties compared with the original, with its formulation presented in Equation (11):

(11) $$x_{n+1}=\mathrm{mod}\left(4x_{n}+0.3-\frac{0.8}{2\pi }\sin\left(2\pi x_{n}\right)+0.25\pi ,1\right)$$

As shown in Figure 1b,d, the scatter plot and frequency histogram for the modified circle chaotic map demonstrate a distribution that exhibits considerably greater uniformity than the original map. Consequently, initializing the DBO with this improved map generates a more diverse initial population with superior spatial coverage.

Based on [40], the strategy of dynamic opposition-based learning (OBL) contributes to a more diverse population, improves the initial solution quality, and accelerates algorithmic convergence. Therefore, this study incorporates the dynamic OBL strategy within the DBO’s initial population generation stage, the mathematical formulation of which is presented in Equation (12):

(12) $$X_{dobl}=X_{init}+r_{1}\times \left(r_{2}\times \left(Lb+Ub-X_{init}\right)-X_{init}\right)$$

In the equation, $X_{init}$ represents the randomly generated initial population, with the variables $r_{1}$ and $r_{2}$ being random numbers sampled from a uniform distribution in the interval [0, 1].

The proposed initialization process, which integrates the improved circle chaotic map and the dynamic opposition-based learning method, proceeds as follows.

Step 1: First, form the starting population A using random initialization. Step 2: Generate a chaotic population B from population A according to Equation (11), and simultaneously generate an opposite population C from population A according to Equation (12). Step 3: Merge populations A, B, and C, and then compute each individual’s fitness score within the newly formed set. Step 4: Sort the resulting fitness values, and then form the final initial population by selecting the top N individuals from the merged group.

### 3.2. Oscillating Balance Factor

Swarm intelligence is fundamentally characterized by a balance between two distinct phases, namely exploration, where the algorithm scans the whole search area for the purpose of discovering potentially optimal regions, and exploitation, where the focus shifts to a more concentrated search within those regions to pinpoint the optimal solution.

**Figure 2:**
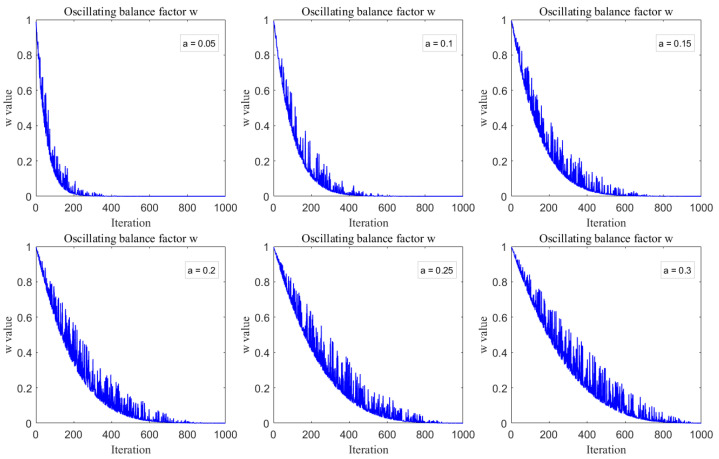
Impact of the parameter α on the variation in *w*.

Global exploration within the DBO framework is the primary responsibility of the ball-rolling dung beetles. To bolster this capability while establishing a more effective trade-off between global search and local refinement, a balance factor, denoted by *w*, which nonlinearly decreases over iterations at a rate adaptively modulated by a sinusoidal function for the purpose of establishing a trade-off between global exploration and local exploitation, is developed and applied to their position update rule, as formulated in Equation (13):

(13) $$w={\left(\frac{T_{max}-t}{T_{max}}\right)}^{\frac{\left|\sin\theta \right|}{\alpha }}$$

wherein, the parameter θ is defined on the interval $\left(\frac{\pi }{6},\frac{\pi }{2}\right)$, while the parameter α serves to control the amplitude of oscillation. Figure 2 illustrates the variation curves of the oscillatory equilibrium factor *w* as a function of different values of α over the course of 1000 iterations.

As illustrated by Equation (13) and Figure 2, the oscillating balance factor *w* gradually declines over the course of the iterations, with a faster rate of decrease in the beginning. Consequently, the algorithm’s step size transitions from large to small over the course of the search, which allows for a more reasonable allocation of resources between its global exploration and local refinement capabilities. Additionally, the sine function, which is parameterized by θ, injects a degree of randomness into the algorithm’s search during the exploration phase. To maintain a proper equilibrium for the oscillating balance factor in both the initial and final stages while also adhering to the principle of avoiding excessively large step sizes in the exploration phase and using the smallest possible step size during the exploitation phase, this study uses a value of 0.2 for the parameter α.

The process of updating their positions is modified by incorporating the oscillating balance factor, *w*, as formulated in Equations (14) and (15):

(14) $$X_{i}\left(t+1\right)=w\times X_{i}\left(t\right)+\alpha \times k\times X_{i}\left(t-1\right)+b\times \Delta x$$

(15) $$X_{i}\left(t+1\right)=w\times X_{i}\left(t\right)+\tan\left(\theta \right)\left|X_{i}\left(t\right)-X_{i}\left(t-1\right)\right|$$

### 3.3. Improved Foraging Strategy

The generation of candidate solutions as part of the DBO’s foraging process is governed by two random numbers: $K_{1}$ and $K_{2}$. A key limitation of this approach is its failure to utilize information from the current best solutions, thereby weakening the algorithm’s local refinement capabilities and slowing its rate of convergence. To address this deficiency, this paper enhances the foraging phase by integrating an optimal value guidance strategy and an adaptive t-distribution perturbation strategy, both of which are incorporated into the position-updating mechanism.

Drawing inspiration from the social learning mechanism in particle swarm optimization (PSO), this study employs an optimal value guidance strategy to accelerate convergence. The core principle of this strategy is to leverage the best-so-far solution to steer the search of subsequent candidates toward the global optimum [41]. By incorporating this strategy within the foraging stage, the position-updating mechanism is reformulated according to Equation (16):

(16) $$\begin{array}{c} X_{i}\left(t+1\right)=X_{i}\left(t\right)+K_{1}\times \left(X_{i}\left(t\right)-Lb^{b}\right)+K_{2}\times \left(X_{i}\left(t\right)-Ub^{b}\right)+\lambda \times \left(X_{gbest}^{l}-X_{i}\left(t\right)\right)\\ \lambda =e^{\frac{t}{M}-1} \end{array}$$

In the equation, λ is the optimal value guidance factor.

The t-distribution [42] (Student’s distribution) is governed by a degree of freedom parameter n. Its two limiting cases are the Cauchy distribution (n=1), which provides strong global search capabilities, and the Gaussian distribution (n→∞), which excels at local exploitation to improve convergence speed [43]. To leverage the complementary strengths of these two behaviors, this study introduces an adaptive t-distribution perturbation strategy. This strategy enables the algorithm to transition from wide-ranging exploration in the initial phases to focused exploitation in the final phases, which serves to enhance the overall convergence speed. Equation (17) provides the specific formula for this position update:

(17) $$\begin{array}{c} X_{new}^{j}=X_{best}^{j}+t\left(iter\right)\times X_{best}^{j}\\ t\left(iter\right)=\cosh{\left(3\times \left(t/M\right)\right)}^{2} \end{array}$$

wherein, the positions of the optimal solution in the *j*th dimension before and after the perturbation are denoted by $X_{best}^{j}$ and $X_{new}^{j}$, respectively. The term t(iter) is a random value sampled from a t-distribution where the degrees of freedom are set by the current iteration number iter.

### 3.4. Multi-Population Differential Co-Evolutionary Mechanism

Despite boosting search efficiency, the original DBO’s position update strategy is susceptible to converging prematurely to local optima. Once trapped in such a state, the algorithm’s search is restricted to a less-than-optimal region of the solution domain, thereby impeding the discovery of the global optimum. The authors of [44] pointed out that this constraint can be addressed by increasing population diversity, a common strategy for which is to introduce mutation operations, among which the differential evolution algorithm has gained attention for its superior search mechanism.Therefore, to strengthen the algorithm’s potential for steering clear of local optima and bolster population diversity, we developed a multi-population differential co-evolutionary mechanism that applies mutation operations to the dung beetle population.

However, a uniform mutation strategy is suboptimal, as it fails to address the diverse evolutionary requirements of all individuals in the population. For example, individuals with high fitness values are usually clustered near the current best individual and thus require more emphasis on the local exploitation capability. In contrast, individuals with inferior fitness are typically distant from the optimal solution, thus requiring a stronger capacity for global exploration. This principle motivates our approach, which involves partitioning the population into three distinct groups, using fitness as the partitioning criterion—the top 20% of the population as the elite group, the middle 50% as the intermediate group, and the remaining 30% as the inferior group—and then applying a unique differential evolution operator to each group. For example, with a total population of 30, the members are allocated accordingly: 6 are assigned to the elite group, 15 are assigned to the intermediate group, and the final 9 form the inferior group. This method enhances the DBO’s overall search effectiveness and facilitates its escape from local optima. The specific strategies are detailed below.

#### 3.4.1. Elite Group

Individuals in the population’s elite group, characterized by high fitness values, are presumed to be in proximity to the global optimum. Consequently, their search should focus on local exploitation—performing fine-grained searches within their immediate region—to pinpoint the optimal solution. To facilitate this, the DE/current-to-best/1 mutation operator is employed for this group. This operator was selected for its exceptional local exploitation capabilities, which align perfectly with the requirements of the elite group, despite its limited global exploration potential. The corresponding mathematical formulation is presented in Equation (18):

(18) $$v_{i}\left(t\right)=x_{i}\left(t\right)+F\times \left[x_{best}\left(t\right)-x_{i}\left(t\right)\right]+F\times \left[x_{r1}\left(t\right)-x_{r2}\left(t\right)\right]$$

wherein, the resulting mutant vector is denoted by $v_{i}\left(t\right)$, while *F* serves as the scaling factor. The best individual in the current generation *t* is represented by $x_{best}\left(t\right)$. The terms $x_{r1}\left(t\right)$ and $x_{r2}\left(t\right)$ represent two separate individuals randomly selected from the population, excluding the target vector $x_{i}$.

Subsequently, a crossover operation derived from differential evolution is applied, with its formulation presented in Equation (19):

(19) $$u_{i,j}\left(t\right)=\left\{\begin{array}{cc} v_{i,j}\left(t\right), & \mathrm{if}\;\mathrm{rand}\left(0,1\right)\leq CR\;\mathrm{or}\;j=j_{rand}\\ x_{i,j}\left(t\right), & \mathrm{otherwise} \end{array}\right.$$

wherein, $j_{rand}$ is an integer selected randomly from the interval [1,D] and CR is the crossover rate parameter. Following this crossover operation, a selection operation is performed using a greedy criterion, as shown in Equation (20):

(20) $$x_{i}\left(t+1\right)=\left\{\begin{array}{cc} u_{i}\left(t\right), & \mathrm{if}\;f\left(u_{i}\left(t\right)\right)\leq f\left(x_{i}\left(t\right)\right)\\ x_{i}\left(t\right), & \mathrm{otherwise} \end{array}\right.$$

#### 3.4.2. Intermediate Group

The intermediate group, which possesses fitness values situated between the superior and inferior extremes, has a dual function, being capable of both local learning from the elite population and global exploration, which assists the algorithm in achieving a more effective trade-off between global search and exploitation. To fulfill the intermediate group’s role of balancing the search, we utilize the DE/mean-current/2 mutation operator, which was selected for its inherent capacity to provide an effective trade-off between global search and local refinement. Equation (21) gives the mathematical definition for this operator:

(21) $$v_{i}\left(t\right)=x_{c1}+F\times \left[x_{c1}-x_{i}\left(t\right)\right]+F\times \left[x_{c2}-x_{i}\left(t\right)\right]$$

wherein, $x_{c1}=\frac{x_{r1}\left(t\right)+x_{r2}\left(t\right)}{2}$, $x_{c2}=\frac{x_{r1}\left(t\right)+x_{best}\left(t\right)}{2}$. After mutation, the crossover operation is performed using Equation (19), and the selection operation is performed using Equation (20).

#### 3.4.3. Inferior Group

Individuals in the inferior group, characterized by poor fitness, are tasked with performing wide-ranging global exploration, which serves to maintain a diverse population and help the algorithm steer clear of local optima. The DE/rand/1 mutation operator is ideally suited for this purpose. By generating perturbations from two random difference vectors, this operator facilitates a broad search of the solution space without relying on guidance from other individuals, thus exhibiting excellent global exploration properties. Its mathematical definition is given by Equation (22):

(22) $$v_{i}\left(t\right)=x_{r1}\left(t\right)+F\times \left[x_{r2}\left(t\right)-x_{r3}\left(t\right)\right]$$

The terms $x_{r1}\left(t\right)$, $x_{r2}\left(t\right)$, and $x_{r3}\left(t\right)$ represent three different individuals, chosen randomly from the existing population. Following the mutation phase, the crossover and selection steps are executed as defined in Equations (19) and (20).

**Figure 3:**
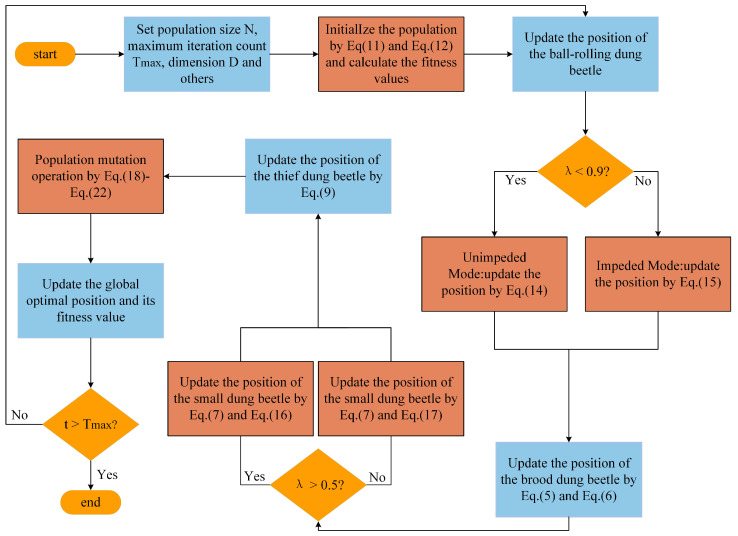
Flowchart of the MIDBO.

The complete operational flow of the MIDBO is detailed in two places: a flowchart presented in Figure 3 and its corresponding pseudocode, given in Algorithm 2.

### 3.5. Time Complexity Analysis of MIDBO

Given a population with a size *N*, a solution space with a dimension *D*, and a maximum iteration count of *M*, the time complexity of the baseline DBO is O(N×D×M). We will now analyze the computational overhead introduced by the strategies integrated into our proposed algorithm:

- Population initialization with the chaotic opposition-based learning strategy: O(N×D+N×logN).
- Oscillating balance factor: O(N×D).
- Improved foraging strategy: O(N×D).
- Multi-population differential co-evolutionary mechanism: O(N×D+N×logN).

Thus, the time complexity of the MIDBO can be expressed as O(M×N×(D+logN)), indicating a rise in computational demands relative to the DBO. However, substantial performance gains for the MIDBO are confirmed through the experiments and real-world engineering cases presented in Section 4 and Section 5. Accordingly, this escalation in complexity is deemed an acceptable compromise in exchange for the algorithm’s augmented efficacy.

**Algorithm 2** The framework of the MIDBO algorithm **Require:** The maximum iteration $T_{max}$, the size of the particle’s population *N*, obtain the initial population *X* of dung beetles by Equations (11) and (12) **Ensure:** Optimal position $X_{best}^{g}$ and its fitness value $f_{min}$     1:    **while **
$t\leq T_{max}$
** do**     2:      **for** i=1 to Number of rolling dung beetles **do**     3:         α=rand(1)     4:         **if** α≤0.9 **then**     5:            Update rolling dung beetle’s position by using Equation (14)     6:         **else**     7:            Update rolling dung beetle’s position by using Equation (15)     8:         **end if**     9:      **end for**  10:      **for** i=1 to Number of breeding dung beetles **do**  11:         Update breeding dung beetle’s position by using Equations (5) and (6)  12:      **end for**  13:      **for** i=1 to Number of small dung beetles **do**  14:         **if** rand>0.5 **then**  15:            Update small dung beetle’s position by using Equations (7) and (16)  16:         **else**  17:            Update small dung beetle’s position by using Equations (7) and (17)  18:         **end if**  19:      **end for**  20:      **for** i=1 to Number of Stealing Dung Beetles **do**  21:         Update stealing dung beetle’s location by using Equation (9)  22:      **end for**  23:      Perform the population mutation operation by using Equations (18)–(22)  24:      t=t+1  25:    **end while**
  26:    **return **$X_{best}^{g}$ and its fitness value $f_{min}$

## 4. Experiments

All experiments in this section were conducted on a conventional personal computer running MATLAB R2024a, configured with a Windows 11 (64-bit) OS, an Intel(R) Core(TM) i9-13900H CPU @ 2.60 GHz, and 24 GB of RAM. To evaluate the performance of the proposed MIDBO on the CEC2017 [45] and CEC2022 [46] test suites, it was compared with the hiking optimization algorithm (HOA) [47], whale optimization algorithm (WOA) [48], differential evolution (DE) [9], particle swarm optimization (PSO) [49], a dung beetle optimization algorithm based on quantum computing and a multi-strategy hybrid (QHDBO) [50], and the original DBO. Both test suites comprise unimodal, multimodal, hybrid, and composition functions, thereby facilitating a more scientific and comprehensive evaluation of the respective merits and demerits of each algorithm. Table 1 lists the specific parameter configurations for all algorithms used in the comparative analysis.

### 4.1. Performance Evaluation of Improved Strategies

Ablation experiments were performed to evaluate how the four integrated strategies, both individually and in combination, affect the DBO’s overall performance. Here, we define the relevant abbreviations used in the experiments: IDBO is DBO + chaotic opposition-based learning; ODBO is the DBO + oscillating balance factor; FDBO is the DBO + improved foraging strategy; DDBO is the DBO + multi-population differential co-evolutionary mechanism; IOFDBO is the DBO + chaotic opposition-based learning + oscillating balance factor + improved foraging strategy; IODDBO is the DBO + chaotic opposition-based learning + oscillating balance factor + multi-population differential co-evolutionary mechanism; IFDDBO is the DBO + chaotic opposition-based learning + improved foraging strategy + multi-population differential co-evolutionary mechanism; OFDDBO is the DBO + oscillating balance factor + improved foraging strategy + multi-population differential co-evolutionary mechanism. The performance of these DBO variants was analyzed using 12 benchmark functions selected from the CEC2017 benchmark set, covering unimodal, simple multimodal, hybrid, and composition functions. For the primary statistical analysis, the canonical DBO was treated as the baseline, which included the mean and ranking. Table 2 summarizes the findings from the ablation experiment.

The analysis of our experiments demonstrates that four new mechanisms, especially the improved foraging strategy and the multi-population differential co-evolutionary mechanism, collectively improved the DBO’s performance. Additionally, it can be seen from the rankings in the last row of the experimental results that IFDDBO, IODDBO, and OFDDBO, which were ranked second, third, and fourth, had better overall optimization performance than the other variants of the DBO. This result underscores the significant contributions of the three main strategies: chaotic opposition-based learning, improved foraging, and multi-population differential co-evolutionary. The MIDBO demonstrated superior performance compared with the DBO, exhibiting enhanced local optima avoidance and improved exploration–exploitation balance. This advancement is a result of the synergistic effect among the four proposed strategies. Together, they effectively guide the algorithm’s search toward the global optimum.

### 4.2. CEC2017 Benchmark Function Results and Analysis

To thoroughly assess the MIDBO’s performance, we benchmarked it against six leading swarm intelligence algorithms using the CEC2017 benchmark suite (29 test functions). This assessment validated MIDBO’s effectiveness and generalizability across diverse optimization problem types. For experimental consistency, we applied identical parameter settings to all algorithms for the population size (N = 30), maximum iterations (T = 1000), and dimensionality (D = 50). To mitigate stochastic variability, for each test function, every algorithm was run independently 30 times, with performance quantified through the mean, standard deviation, and ranking.

With an average rank of 1.1379 across the 29 CEC2017 test functions, the MIDBO achieved the highest overall rank, a finding clearly supported by the results in Table 3. Notably, MIDBO significantly outperformed the original DBO across all test functions, a result that validates the effectiveness of our proposed modifications. The MIDBO ranked first and recorded the best mean value when its performance was analyzed on unimodal functions F1 and F3. The MIDBO also obtained the best mean and rank on multimodal functions F4, F5, F8, and F10. However, it did not secure the best mean on F6 and F7, ranking third. On F9, the MIDBO’s mean and rank were inferior to PSO’s, placing it second. In the hybrid and composition functions, the MIDBO’s mean on F27 was inferior to that of the DE algorithm; however, despite not achieving the optimal mean, it still ranked first. For all functions other than these, it obtained the best mean and the first rank.

Table 3 presents an analysis of the 50-dimensional problem results on the CEC2017 benchmark, revealing a distinct performance hierarchy among the compared algorithms. The MIDBO secured the premier rank, surpassing the QHDBO, DBO, and HOA, which ranked second, third, and last, respectively. These results provide compelling evidence that the novel strategies implemented in the MIDBO are highly effective at enhancing the convergence precision of the baseline DBO. The seven algorithms were ranked by performance in the following order: MIDBO > QHDBO > DBO > DE > PSO > WOA > HOA.

The results obtained from the Wilcoxon rank-sum test are displayed in Table 4. A *p* value exceeding 0.05, highlighted in bold, indicates the absence of a statistically significant difference and is denoted by the symbol “=”. Conversely, a *p* value below 0.05 signals a significant performance disparity. In such instances, superiority was assigned to the algorithm with the lower mean value, where “+” signifies that the MIDBO is superior while “-” indicates the superiority of the comparison algorithm. Adhering to this convention, the summary tallies of the MIDBO’s performance against each competitor were 29/0/0, 21/8/0, 25/4/0, 29/0/0, 25/3/1, and 22/6/1. In the comparison between the MIDBO and DBO, *p* > 0.05 on functions F7, F10, F18, and F22, and the MIDBO’s performance was superior to the DBO’s on the remaining 25 functions. Compared with DE, *p* > 0.05 on functions F6, F14, F15, F23, F24, F26, F27, and F29, and the MIDBO’s performance was superior to DE’s on the remaining 21 functions. Compared with PSO, *p* > 0.05 on functions F9, F26, and F27. On function F6, PSO was superior to the MIDBO, but the MIDBO’s performance was superior to PSO’s on the remaining 25 functions. Compared with the QHDBO, *p* > 0.05 on functions F6, F10, F14, F18, F25, and F26. As the QHDBO achieved the optimal mean value on function F7, it was considered superior on this function. However, the MIDBO’s performance was superior to the QHDBO’s on the remaining 22 functions. A comparative analysis with the two other algorithms demonstrates that the MIDBO outperformed all of them. Consequently, the MIDBO exhibited superior capability in solving 50-dimensional CEC2017 benchmark functions.

**Figure 4:**
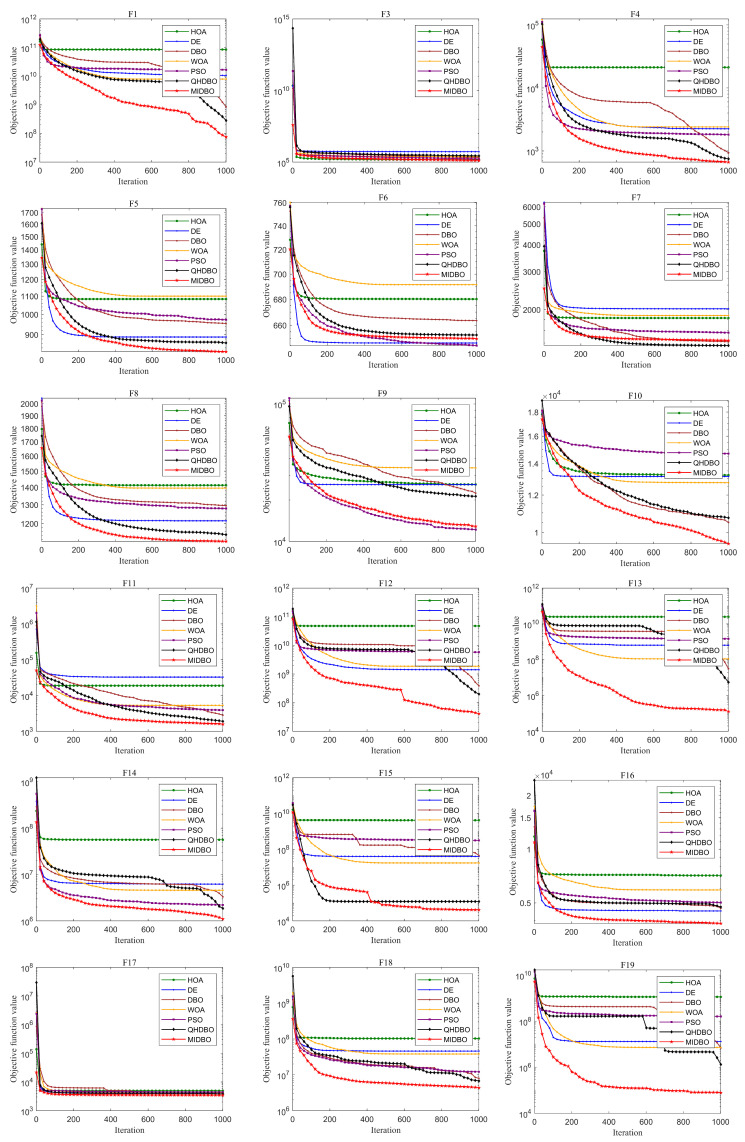

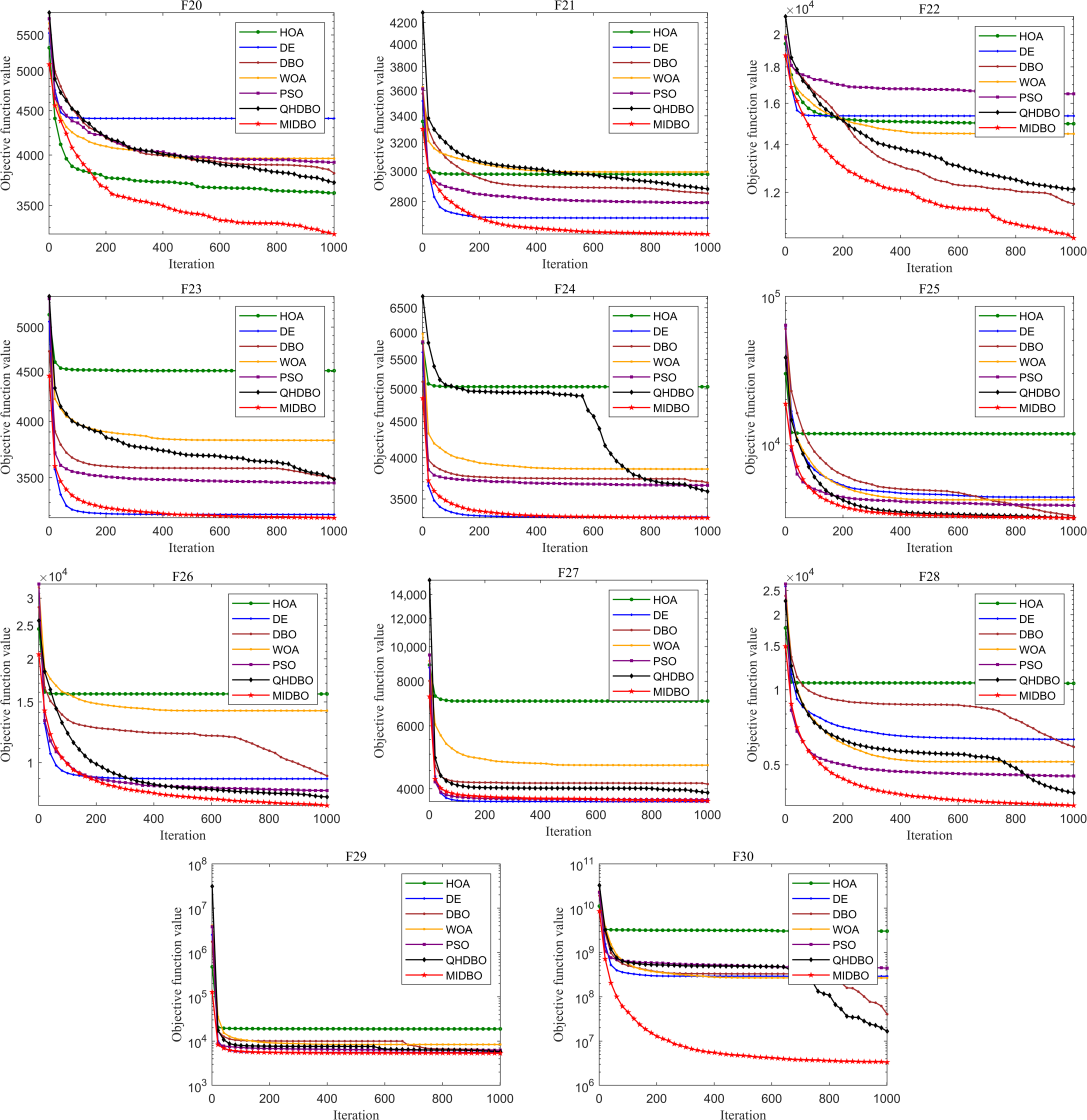
CEC 2017 convergence curves.

Figure 4 presents how all algorithms converged for the CEC2017 test suite. For unimodal functions, the MIDBO’s convergence performance demonstrated substantial superiority over the competing algorithms. The MIDBO exhibited slower early-stage convergence than the competing algorithms for multimodal and hybrid functions. However, the incorporation of an enhanced foraging strategy and a multi-population differential cooperative evolution mechanism provides the algorithm with a more effective mechanism for circumventing local optima. Therefore, as the iterations progressed, after other algorithms already stagnated and converged to a suboptimal solution, the MIDBO’s convergence curve still showed a downward trend, and in terms of convergence accuracy, the MIDBO clearly outperformed its competitors. The MIDBO demonstrated a particularly strong performance on functions F4, F5, F10–F16, F18 and F19. Its optimization capabilities were also more prominent on the composition functions, specifically F20–F23, F26, F28, and F30, when compared with other algorithms.

**Figure 5:**
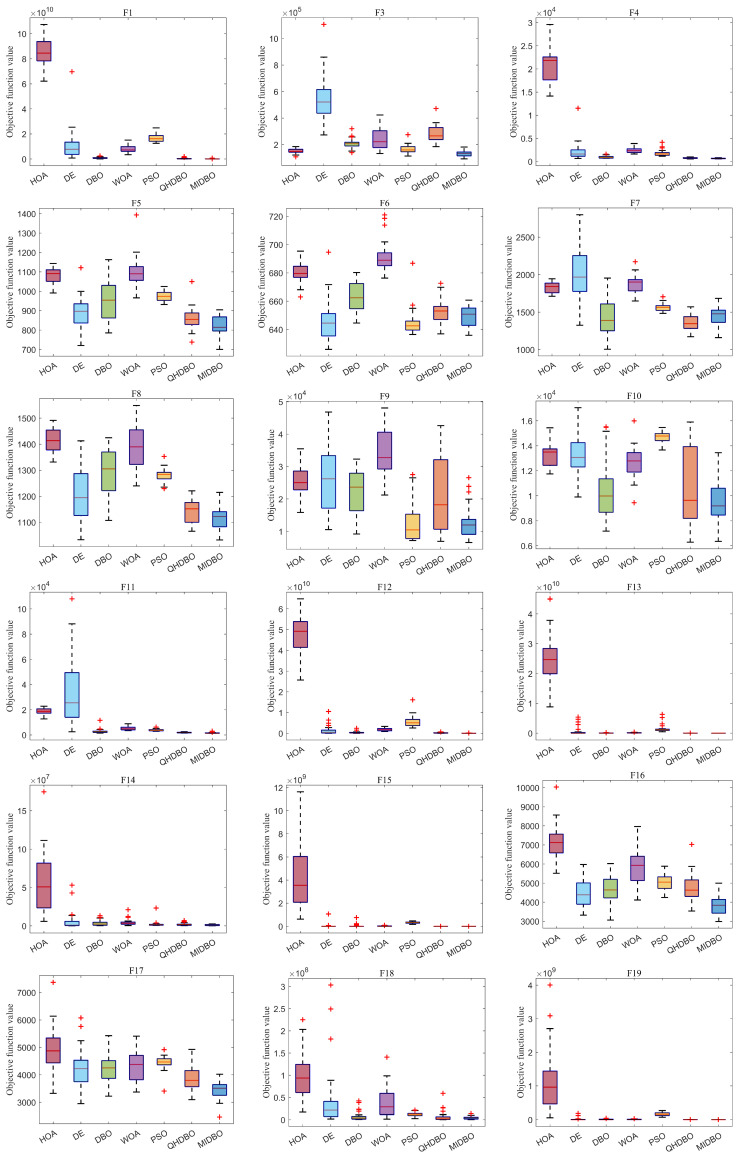

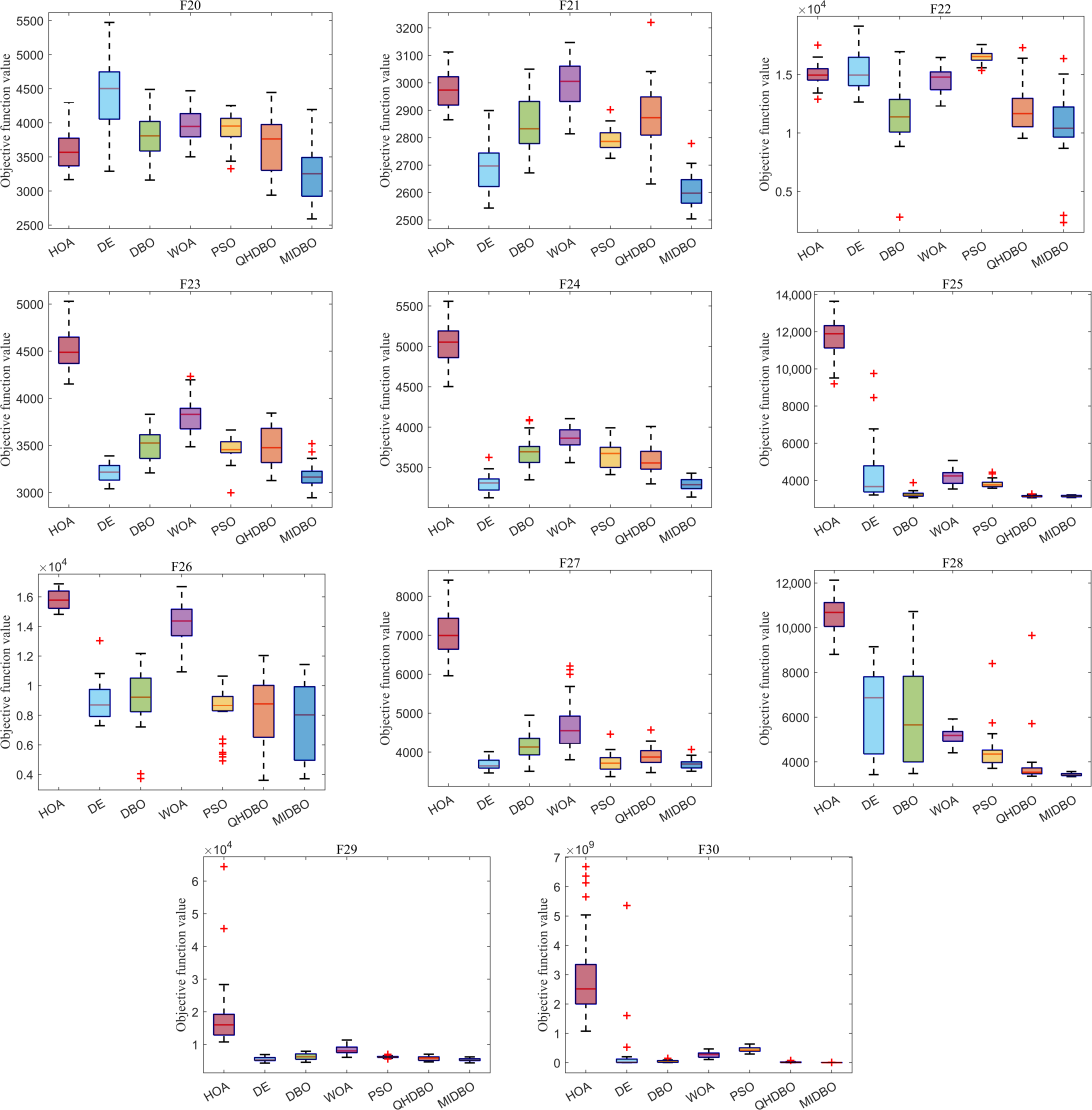
CEC2017 box plots, where “+” denotes outliers.

Figure 5 presents the box plot analysis of algorithm performance on the CEC2017 benchmark suite. In most function cases, the MIDBO exhibited significantly smaller and lower quartile ranges, demonstrating both superior solution quality and enhanced stability relative to the competing methods. When set against the other algorithms, the MIDBO algorithm’s boxes were consistently smaller and positioned lower, especially on functions F1, F3–F4, F11–F16, F18–F19, F23–F25, and F28–F30. Overall, the box plots indicate that the MIDBO showed a significant improvement compared with the DBO.

**Figure 6:**
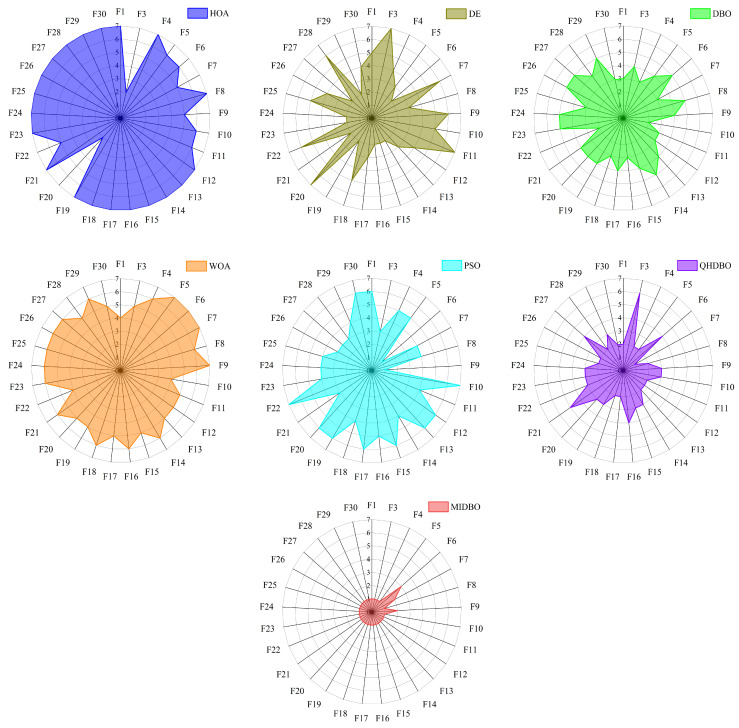
Radar chart for the CEC2017 test suite.

Figure 6 displays the radar chart comparing the ranking performance of the seven algorithms across all 29 functions. The radar chart analysis revealed that the MIDBO achieved the minimal enclosed area among all algorithms, indicating superior overall performance. Conversely, the HOA demonstrated the maximal area, while the DBO ranked third in terms of the enclosed space magnitude. These results demonstrate that the implemented enhancement strategies substantially improved the DBO’s performance while showing the MIDBO’s overall superiority over all comparative algorithms.

### 4.3. CEC2022 Benchmark Function Results and Analysis

To conduct a more comprehensive evaluation of the MIDBO’s effectiveness in solving complex optimization problems, we performed a systematic comparative analysis between the MIDBO and other selected algorithms using the CEC2022 benchmark suite. The experimental parameters were set as follows: a population size of 30, termination criterion of 1000 iterations, and 30 replicates to ensure result robustness. The comprehensive performance evaluation, supported by statistical analysis of the experimental data, highlights the marked advantage of the MIDBO (Table 5). It achieved a first-place finish among all compared algorithms with an average rank of 1.5, a result that corroborates its potent capabilities in global optimization. The performance advantage of the MIDBO became particularly pronounced when evaluated on specific test functions. Specifically, on eight of the benchmark functions (F1, F2, F4, F6–F8, F11, and F12), the MIDBO delivered the best average convergence accuracy while consistently ranking first. This consistency indicates the algorithm’s proficiency in maintaining an efficient optimization process across problems with diverse complexities. An exception occurred on function F10, where the algorithm secured the third rank. Furthermore, on functions F3 and F9, the MIDBO failed to obtain the optimal mean and rank, placing it third behind the DBO and QHDBO. On function F5, although the MIDBO’s mean was marginally inferior to PSO’s, it still achieved the first rank. While there were minor fluctuations in performance on these few functions, the overall findings decisively confirm the effectiveness of the MIDBO in improving convergence accuracy.

According to Table 5, the seven algorithms’ performance on the CEC2022 benchmark can be summarized with the following ranking: MIDBO > DBO > QHDBO > DE > PSO > WOA > HOA.

A Wilcoxon rank sum test was conducted, with the findings detailed in Table 6. The specific win/tie/loss results were 12/0/0, 9/3/0, 6/5/1, 11/1/0, 11/1/0, and 8/3/1. In the comparison between the MIDBO and DBO, *p* > 0.05 was observed on functions F3–F5, F7, and F10. On function F9, the DBO’s performance was superior to the MIDBO’s. Nevertheless, the MIDBO outperformed the DBO on the remaining six functions. Compared with DE, *p* > 0.05 was observed on functions F2, F3, and F4, while the MIDBO was superior on the other nine functions. Similarly, in the comparisons with the WOA and PSO, *p* > 0.05 was observed on functions F6 and F5, respectively, with the MIDBO proving superior results on the remaining 11 functions in each case. Against the QHDBO, *p* > 0.05 was observed on functions F3, F8, and F11. The QHDBO was superior only on function F9, whereas the MIDBO held an advantage on the other eight functions. Finally, the MIDBO exhibited a comprehensive superiority over the HOA. Consequently, it achieved more competitive results on the CEC2022 benchmark.

**Figure 7:**
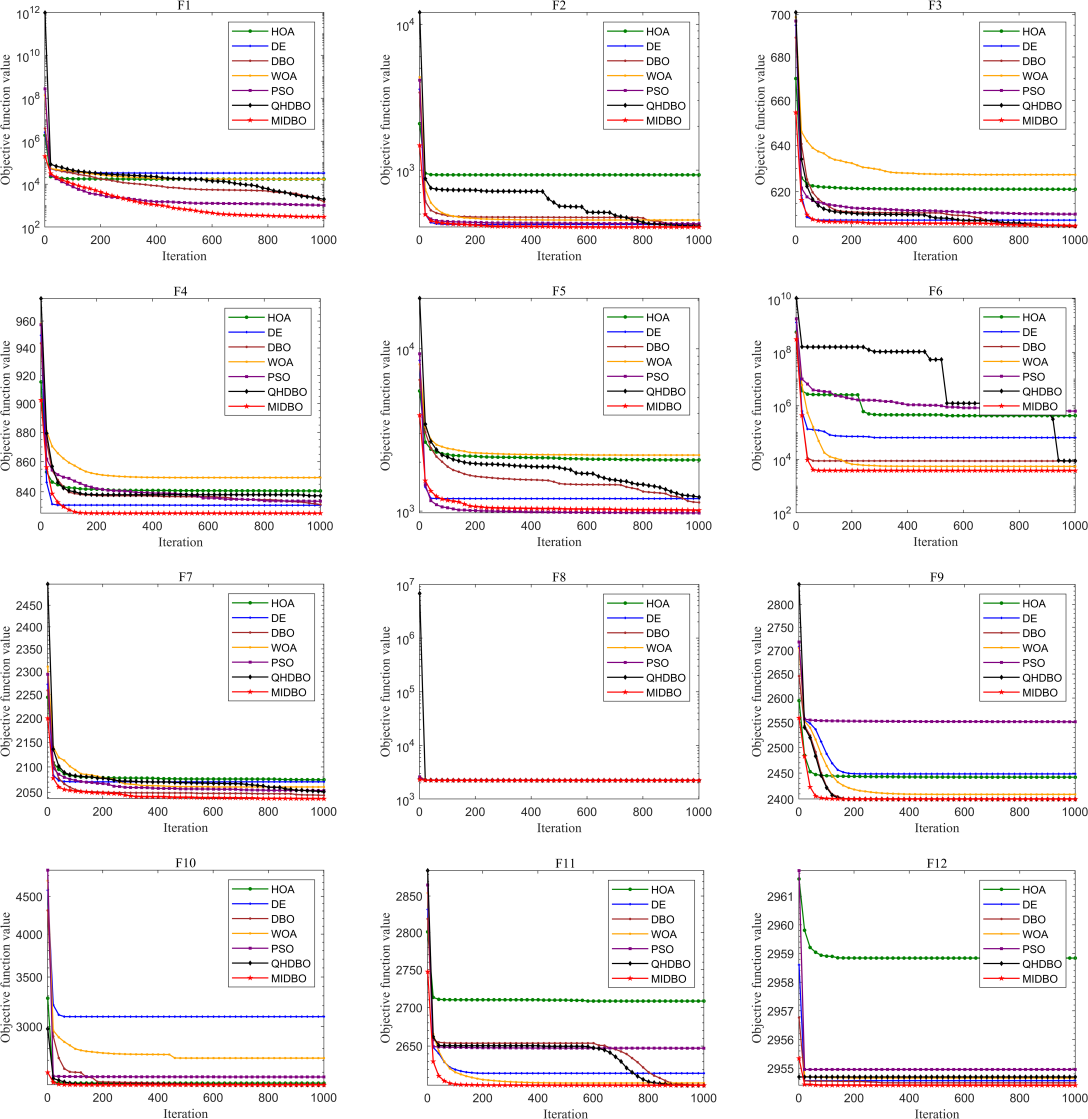
CEC2022 convergence curves.

Figure 7 presents how all algorithms converged for the CEC2022 test suite. On function F1, its convergence speed was initially slower than PSO’s. However, as the iterations progressed, PSO plateaued, while the MIDBO maintained its downward trend, ultimately reaching the optimal value. On function F5, the MIDBO’s initial convergence was surpassed by both DE and PSO. In the subsequent stages of the run, while DE’s rate of descent began to stagnate, the MIDBO maintained a consistent downward trajectory. Nevertheless, its convergence rate remained slower than that of PSO, and it ultimately failed to match the final precision achieved by the latter. Regarding function F9, the MIDBO initially outpaced all competing algorithms, but its progress began to plateau in the later stages, eventually reaching a final accuracy comparable to that of the DBO and QHDBO. The MIDBO demonstrated a superior rate of convergence and, in most instances, higher solution precision, with the exception of a few where it was either slower or failed to reach the optimal value. This robust performance affirms the efficacy of the multiple strategies, as their synergy is what empowers the algorithm to converge on the optimal optimum.

**Figure 8:**
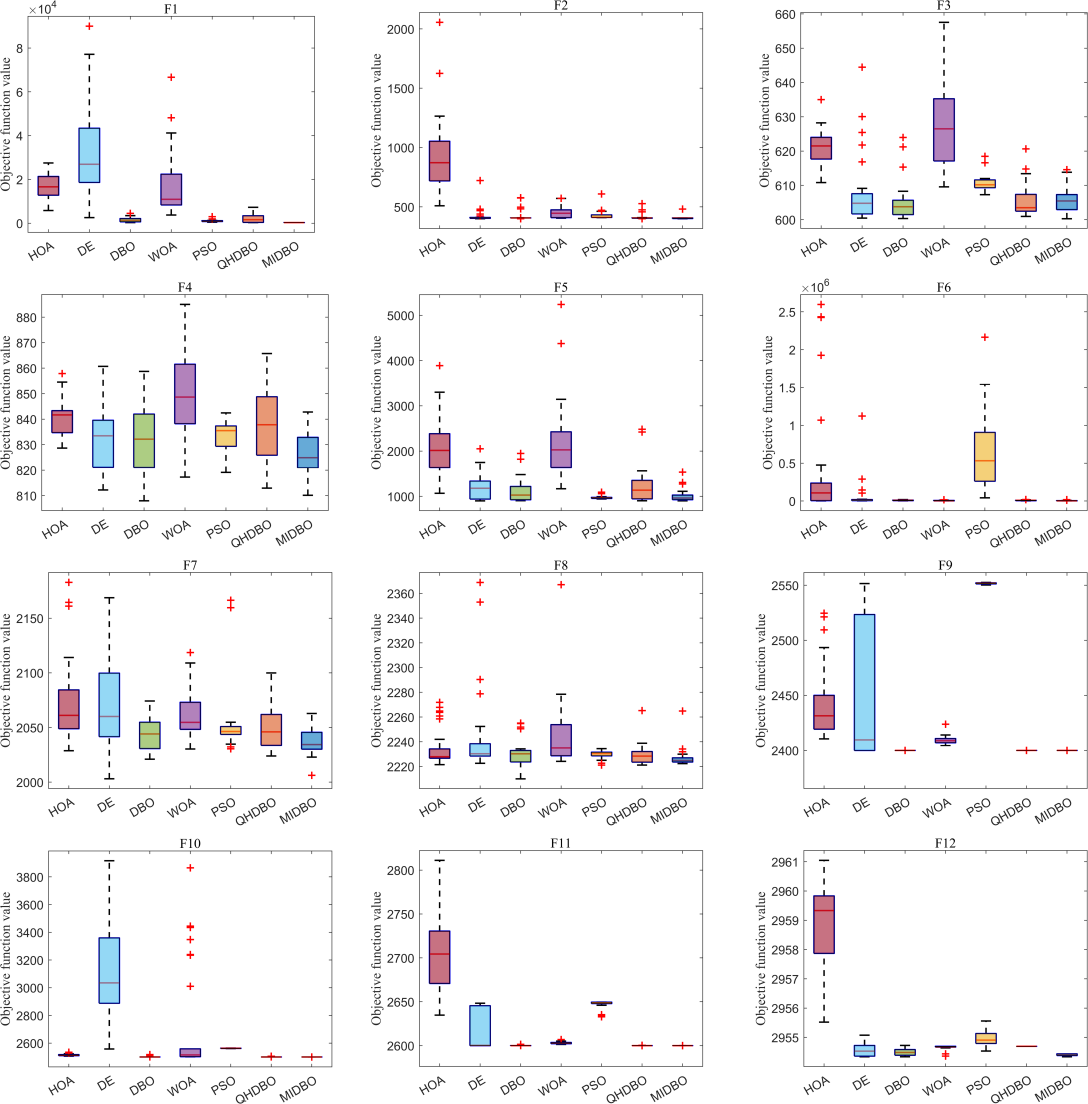
CEC2022 box plots, where “+” denotes outliers.

Using box plots, Figure 8 presents a comparative analysis of the algorithms’ effectiveness on the CEC2022 benchmark. Figure 8 illustrates that the MIDBO’s boxes were smaller and positioned lower on most functions, indicating its superior performance. For functions F3, F4, and F7, while the MIDBO exhibited a larger box size than PSO, its box was positioned lower. On F5, the MIDBO’s overall performance was inferior to PSO’s. On function F4, the MIDBO’s box was also larger than that of the HOA, but it maintained a lower position. In summary, the collective results establish the MIDBO’s clear superiority over its counterparts, and this outcome, supported by the box plot analysis, powerfully underscores the value of the enhancements presented in this paper.

**Figure 9:**
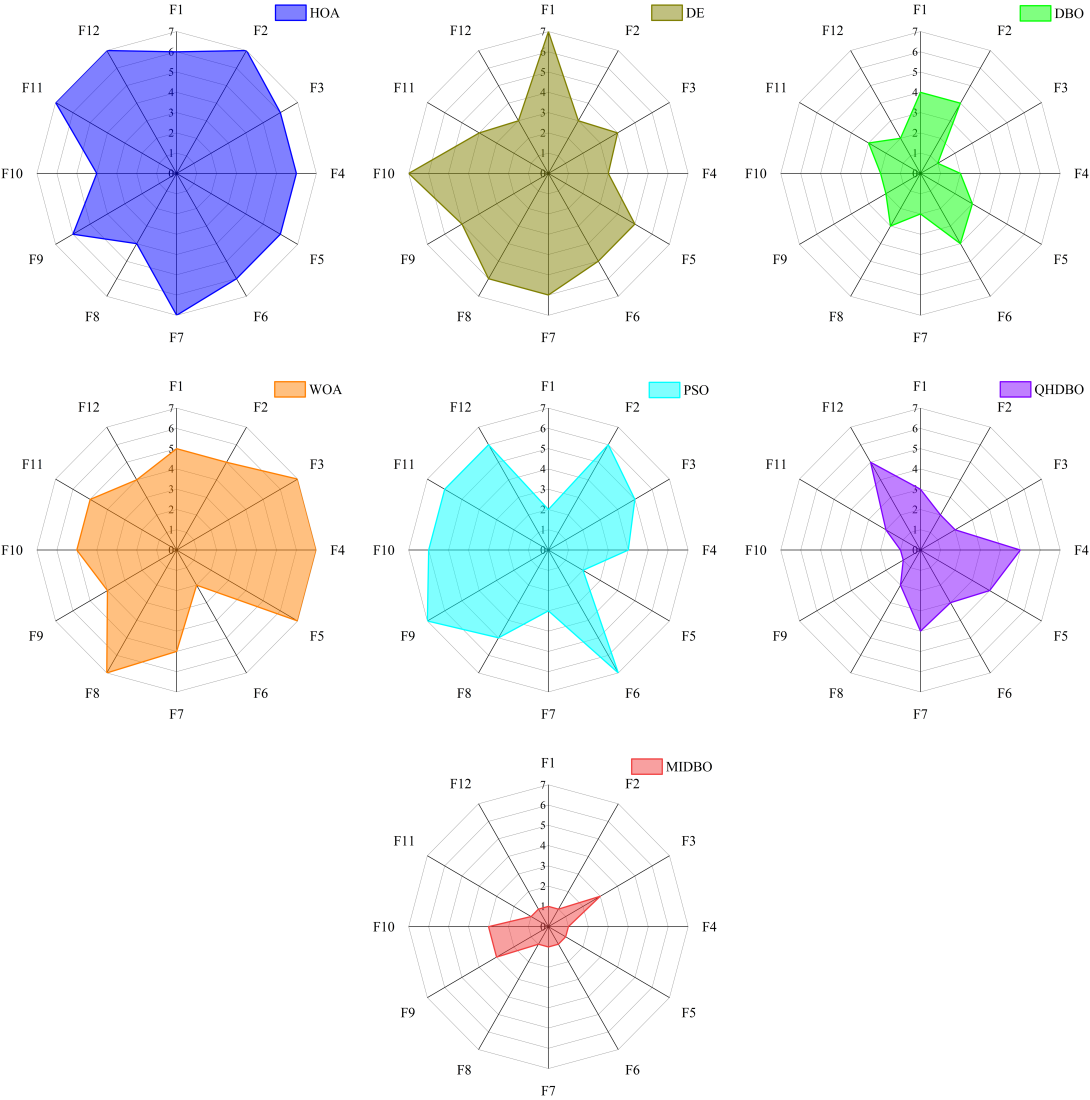
Radar chart for the CEC2022 test suite.

Figure 9 displays the radar chart, which compares the ranks achieved by the seven algorithms for the 12 test functions from the CEC2022 suite. The figure reveals that the MIDBO’s enclosed shape had the smallest area. In contrast, the HOA’s area was the largest, while the DBO ranked second and the QHDBO ranked third. The radar chart’s enclosed areas confirm that the various proposed strategies notably improved the DBO’s optimization performance.

## 5. Engineering Optimization Issues

The effectiveness of the MIDBO in real-world scenarios is examined in this section. To accomplish this, its performance was validated on three standard engineering design case studies. The three issues included the tension–compression spring design problem [51], the pressure vessel design problem [52], and the speed reducer design problem [53]. Due to the multi-constraint nature of these engineering problems, because of its simplicity and straightforward application, this study utilized the penalty function method to manage the problem’s constraints. The function is formulated as shown below:

(23) $$F\left(\vec{x}\right)=f\left(\vec{x}\right)+r\cdot \left[\sum_{i=1}^{m}{\left(\max\left(0,g_{i}\left(\vec{x}\right)\right)\right)}^{\gamma }+\sum_{j=1}^{n}\left(\right|h_{j}\left(\vec{x}\right){\left|\right)}^{\eta }\right]$$

wherein, the modified objective function is denoted by $F(\vec{x})$. The experiments were conducted with the penalty coefficient *r* set to 10e100. The terms $g_{i}\left(\vec{x}\right)$ and $h_{j}\left(\vec{x}\right)$ correspond to the functions for the inequality and equality constraints, respectively, and γ and η are constants, which were set to two in this paper. The total counts of the inequality and equality constraint functions are denoted by *m* and *n*, respectively. A penalty function works by augmenting the objective function’s value with a penalty term in the event of a constraint violation by a candidate solution, thereby directing the algorithm’s search toward more promising and feasible regions.

For the engineering design problem experiments, the same set of comparative algorithms as that in the preceding section was used. The experimental parameters were configured as follows: a population size of 30, termination criterion of 500 iterations, and 20 replicates to ensure result robustness. Performance was evaluated using four statistical metrics: the best, mean, standard deviation, and worst values.

### 5.1. Tension–Compression Spring Design Issues

The classic tension–compression spring design challenge involves optimizing three key variables: the wire diameter (*d*), mean coil diameter (*D*), and number of active coils (*N*). The primary objective is to find the design with the lowest possible weight while adhering to constraints on the minimum deflection, surge frequency, and shear stress. The governing equations for this task are presented below in Equations (24)–(27).

Consider

(24) $$\vec{x}=\left[x_{1},x_{2},x_{3}\right]=\left[d,D,P\right]$$

Minimize

(25) $$f\left(\vec{x}\right)=\left(x_{3}+2\right)x_{2}x_{1}^{2}$$

subject to

(26) $$\left.\begin{array}{cc} g_{1}\left(\vec{x}\right) & =1-\frac{x_{2}^{3}x_{3}}{71,\;785x_{1}^{4}}\leq 0\\ g_{2}\left(\vec{x}\right) & =\frac{4x_{2}^{2}-x_{1}x_{2}}{12,\;566(x_{2}x_{1}^{3}-x_{1}^{4})}+\frac{1}{5108x_{1}^{2}}\leq 0\\ g_{3}\left(\vec{x}\right) & =1-\frac{140.45x_{1}}{x_{2}^{2}x_{3}}\leq 0\\ g_{4}\left(\vec{x}\right) & =\frac{x_{1}+x_{2}}{1.5}-1\leq 0 \end{array}\right\}$$

The variable range is

(27) $$0.05\leq x_{1}\leq 2,\;0.25\leq x_{2}\leq 1.3,\;2\leq x_{3}\leq 15.$$

The findings, summarized in Table 7 and Table 8, reveal that the MIDBO outperformed all other algorithms on this problem by achieving superior optimization accuracy and significantly better stability. The algorithm’s high ranking underscores its strong competitive edge when tackling the tension–compression spring design challenge.

### 5.2. Pressure Vessel Design Issues

The pressure vessel design challenge required optimizing four key parameters: the shell thickness ($x_{1}$), head thickness ($x_{2}$), inner radius ($x_{3}$), and the vessel’s length (excluding heads) ($x_{4}$). Minimizing the total cost of the vessel while satisfying four specific constraints was the main goal of this design problem. The governing mathematical model for this task is provided in Equations (28)–(31).

Consider

(28) $$\vec{x}=\left[x_{1},x_{2},x_{3},x_{4}\right]=\left[T_{s},T_{h},R,L\right]$$

Minimize

(29) $$f\left(\vec{x}\right)=1.7781x_{2}x_{3}^{2}+0.6224x_{1}x_{3}x_{4}+3.1661x_{1}^{2}x_{4}+19.84x_{1}^{2}x_{3}$$

subject to

(30) $$\left.\begin{array}{cc} g_{1}\left(\vec{x}\right) & =-x_{1}+0.0193x_{3}\leq 0,\\ g_{2}\left(\vec{x}\right) & =-x_{2}+0.00954x_{3}\leq 0,\\ g_{3}\left(\vec{x}\right) & =x_{4}-240\leq 0,\\ g_{4}\left(\vec{x}\right) & =-\pi x_{3}^{2}x_{4}-\frac{4}{3}\pi x_{3}^{3}+1296000\leq 0 \end{array}\right\}$$

With a variable range

(31) $$1\leq x_{1},x_{2}\leq 99,\;10\leq x_{3},x_{4}\leq 200.$$

Table 9 and Table 10 provide a performance assessment of seven algorithms applied to the pressure vessel design challenge. The experimental outcomes unequivocally establish the significant advantages of the proposed MIDBO concerning its optimization accuracy and average performance. To be specific, the MIDBO outperformed all competitors in the two key metrics of the best and mean values, securing an optimal value of 5743.021200 and an average value of 6033.448733. Notably, the PSO algorithm exhibited superior performance in the worst value and Std metrics. Nonetheless, the MIDBO’s premier optimization capability and exceptional average performance confirm its enhanced overall efficacy and practical value for tackling engineering challenges characterized by complex constraints and stringent precision demands, setting it apart from the other compared algorithms.

### 5.3. Speed Reducer Design Issues

The speed reducer design issues involve optimizing a set of seven parameters: the face width ($b=x_{1}$), module of teeth ($m=x_{2}$), number of teeth on the pinion ($p=x_{3}$), lengths of the first and second shafts between bearings ($l_{1}=x_{4}$ and $l_{2}=x_{5}$, respectively), and the diameters of the first and second shafts ($d_{1}=x_{6}$ and $d_{2}=x_{7}$, respectively). Finding the variable combination that yielded the lowest possible weight for the speed reducer was the primary objective, all while adhering to 11 engineering constraints. The governing equations for this task are provided in Equations (32)–(35).

Consider

(32) $$\vec{x}=\left[x_{1},x_{2},x_{3},x_{4},x_{5},x_{6},x_{7}\right]=\left[b,m,p,l_{1},l_{2},d_{1},d_{2}\right]$$

Minimize

(33) $$\begin{array}{cc} f(\vec{x})= & 0.7854x_{1}x_{2}^{2}\left(3.3333x_{3}^{2}+14.9334x_{3}-43.0934\right)\\ \; & -1.508x_{1}\left(x_{6}^{2}+x_{7}^{2}\right)+0.7854x_{1}\left(x_{4}x_{6}^{2}-x_{5}x_{7}^{2}\right) \end{array}$$

subject to

(34) $$\left.\begin{array}{cc} g_{1}\left(\vec{x}\right) & =\frac{27}{x_{1}x_{2}^{2}x_{3}}-1\leq 0,\\ g_{2}\left(\vec{x}\right) & =\frac{397.5}{x_{1}x_{2}^{2}x_{3}^{2}}-1\leq 0,\\ g_{3}\left(\vec{x}\right) & =\frac{1.93x_{4}^{3}}{x_{2}x_{6}^{4}x_{3}}-1\leq 0,\\ g_{4}\left(\vec{x}\right) & =\frac{1.93x_{5}^{3}}{x_{2}x_{7}^{4}x_{3}}-1\leq 0,\\ g_{5}\left(\vec{x}\right) & =\frac{\sqrt{{\left(\frac{745x_{4}}{x_{2}x_{3}}\right)}^{2}+16.9\times 10^{6}}}{110x_{6}^{3}}-1\leq 0,\\ g_{6}\left(\vec{x}\right) & =\frac{\sqrt{{\left(\frac{745x_{5}}{x_{2}x_{3}}\right)}^{2}+157.5\times 10^{6}}}{85x_{7}^{3}}-1\leq 0,\\ g_{7}\left(\vec{x}\right) & =\frac{x_{2}x_{3}}{40}-1\leq 0,\\ g_{8}\left(\vec{x}\right) & =\frac{5x_{2}}{x_{1}}-1\leq 0,\\ g_{9}\left(\vec{x}\right) & =\frac{x_{1}}{12x_{2}}-1\leq 0,\\ g_{10}\left(\vec{x}\right) & =\frac{1.5x_{6}+1.9}{x_{4}}-1\leq 0,\\ g_{11}\left(\vec{x}\right) & =\frac{1.1x_{7}+1.9}{x_{5}}-1\leq 0 \end{array}\right\}$$

With a variable range of

(35) $$\begin{array}{cc} \; & 2.6\leq x_{1}\leq 3.6,\;0.7\leq x_{2}\leq 0.8,\;17\leq x_{3}\leq 28,\;7.3\leq x_{4}\leq 8.3,\\ \; & 7.3\leq x_{5}\leq 8.3,\;2.9\leq x_{6}\leq 3.9,\;5\leq x_{7}\leq 5.5 \end{array}$$

As demonstrated by the data in Table 11 and Table 12, the different algorithms exhibited varied performance in terms of optimization accuracy and stability. Although the DE algorithm achieved the best value (2513.700952), its standard deviation (75.53042354) was fourth among all compared algorithms, indicating that its results were highly unstable. In contrast, our proposed MIDBO demonstrated an exceptional balance in its performance. It not only secured the second-best optimization accuracy (2994.234252), outperforming most of the comparative algorithms, including the baseline DBO, but more importantly, it achieved the best stability with a standard deviation of 0.000319158, far surpassing all other algorithms. Compared with the baseline DBO, the MIDBO showed significant improvements across all four metrics. In conclusion, the MIDBO, while preserving a high-accuracy solution capability, greatly enhanced algorithmic stability, rendering it an efficient and highly reliable option for solving such engineering optimization problems.

## 6. Conclusions

In this paper, we proposed an improved Dung Beetle Optimizer (MIDBO), which adds a chaotic opposition-based learning strategy, an oscillating balance factor strategy, an improved foraging strategy, and a multi-population differential co-evolutionary mechanism to the DBO. This improved algorithm was benchmarked against the original DBO and other swarm intelligence algorithms using the CEC2017 and CEC2022 benchmark suites to assess its performance. The MIDBO, as shown by the experimental results, excelled in two key areas: its speed of convergence and the precision of its solutions. Furthermore, the results from three real-world engineering challenges confirmed MIDBO’s strong performance when handling complex optimization tasks.

Despite the MIDBO’s overall strong performance, numerical experiments revealed its relatively poor convergence accuracy on certain functions across the two test suites, suggesting that there is still room for further enhancement. The complexity of the MIDBO has increased due to the addition of multiple strategies. Therefore, future work could involve combining the proposed MIDBO with other swarm intelligence algorithms to form more effective hybrid algorithms to further improve its performance. To enrich the international scope of this research and serve as an impetus for the algorithm’s continued evolution, our future efforts will focus on a comparative analysis involving advanced algorithms drawn from the global literature. This approach is designed to remedy the constrained range of our current reference works. The initiative aims to ensure that the MIDBO’s performance is evaluated against internationally recognized benchmarks and furnish a more resilient and diversified theoretical underpinning for its subsequent refinement.

Additionally, while the MIDBO has demonstrated commendable performance on engineering design problems, according to the NFL theorem, there is no universally optimal algorithm suited for all optimization challenges. Consequently, the extent of the MIDBO’s competitive edge in more arduous engineering domains warrants further investigation. This recognition impels our future work toward probing the applicability of the MIDBO in sophisticated engineering contexts—such as unmanned aerial vehicle path planning and task allocation, regression prediction, feature selection, and the optimization of model parameters—with the ultimate goal of broadening its range of practical applications. We hope that these studies can contribute to the development of the swarm intelligence field by providing more efficient methods and tools.

## Figures and Tables

**Table 1:** Algorithm parameters.

Algorithm	Parameter	Value
HOA	Angle of inclination $\theta_{i,t}$	[0, $50^{\circ }$]
	Sweep factor	[1,3]
WOA	*a*	[2,0]
	*b*	1
DE	*F*	0.7
	CR	0.85
DBO	*k*	0.1
	*b*	0.3
	*S*	0.5
PSO	$c_{1},c_{2}$	2
QHDBO	*RDB*	6
	*EFDB*	13
	*SDB*	11
MIDBO	The same as the DBO parameters	

**Table 2:** Ablation experiment results.

Functions	DBO	IDBO	ODBO	FDBO	DDBO	IOFDBO	IODDBO	IFDDBO	OFDDBO	MIDBO
F3	9.1480 $\times \;10^{04}$	8.8074 $\times \;10^{04}$	9.0500 $\times \;10^{04}$	7.2175 $\times \;10^{04}$	5.2573 $\times \;10^{04}$	7.1975 $\times \;10^{04}$	4.9882 $\times \;10^{04}$	5.1352 $\times \;10^{04}$	5.2631 $\times \;10^{04}$	**4.9674 $\times \;10^{04}$**
F9	5.8306 $\times \;10^{03}$	6.1156 $\times \;10^{03}$	5.3464 $\times \;10^{03}$	4.6291 $\times \;10^{03}$	3.8974 $\times \;10^{03}$	4.7167 $\times \;10^{03}$	3.8323 $\times \;10^{03}$	4.1896 $\times \;10^{03}$	3.7567 $\times \;10^{03}$	**3.5808 $\times \;10^{03}$**
F10	6.5226 $\times \;10^{03}$	6.3232 $\times \;10^{03}$	6.5760 $\times \;10^{03}$	6.0762 $\times \;10^{03}$	5.9728 $\times \;10^{03}$	6.1474 $\times \;10^{03}$	6.3496 $\times \;10^{03}$	**5.6195 $\times \;10^{03}$**	5.8266 $\times \;10^{03}$	5.7701 $\times \;10^{03}$
F11	1.8481 $\times \;10^{03}$	1.5606 $\times \;10^{03}$	1.9749 $\times \;10^{03}$	1.3736 $\times \;10^{03}$	1.3377 $\times \;10^{03}$	1.3722 $\times \;10^{03}$	1.3187 $\times \;10^{03}$	1.3266 $\times \;10^{03}$	1.3145 $\times \;10^{03}$	**1.2949 $\times \;10^{03}$**
F13	1.3558 $\times \;10^{07}$	1.0774 $\times \;10^{07}$	2.1910 $\times \;10^{07}$	2.1798 $\times \;10^{06}$	1.2350 $\times \;10^{05}$	1.3423 $\times \;10^{05}$	1.5383 $\times \;10^{05}$	3.4804 $\times \;10^{05}$	1.7070 $\times \;10^{05}$	**1.1127 $\times \;10^{05}$**
F15	2.6815 $\times \;10^{05}$	5.0277 $\times \;10^{04}$	8.1435 $\times \;10^{05}$	1.8088 $\times \;10^{07}$	2.7319 $\times \;10^{04}$	2.7360 $\times \;10^{04}$	1.3875 $\times \;10^{04}$	1.6778 $\times \;10^{04}$	1.3703 $\times \;10^{04}$	**1.2670 $\times \;10^{04}$**
F16	3.3652 $\times \;10^{03}$	3.3148 $\times \;10^{03}$	3.3964 $\times \;10^{03}$	3.0473 $\times \;10^{03}$	2.9245 $\times \;10^{03}$	2.9599 $\times \;10^{03}$	2.8980 $\times \;10^{03}$	2.8956 $\times \;10^{03}$	2.9186 $\times \;10^{03}$	**2.8847 $\times \;10^{03}$**
F17	2.6582 $\times \;10^{03}$	2.4675 $\times \;10^{03}$	2.6686 $\times \;10^{03}$	2.5544 $\times \;10^{03}$	2.4240 $\times \;10^{03}$	2.5031 $\times \;10^{03}$	2.3827 $\times \;10^{03}$	2.3913 $\times \;10^{03}$	2.4043 $\times \;10^{03}$	**2.3716 $\times \;10^{03}$**
F19	2.3895 $\times \;10^{06}$	6.9655 $\times \;10^{06}$	4.0966 $\times \;10^{06}$	5.1020 $\times \;10^{04}$	2.9648 $\times \;10^{04}$	4.9796 $\times \;10^{04}$	1.8855 $\times \;10^{04}$	2.8460 $\times \;10^{04}$	2.3722 $\times \;10^{04}$	**1.1785 $\times \;10^{04}$**
F22	5.4224 $\times \;10^{03}$	2.6580 $\times \;10^{03}$	5.0387 $\times \;10^{03}$	3.2489 $\times \;10^{03}$	4.0564 $\times \;10^{03}$	2.3804 $\times \;10^{03}$	3.6102 $\times \;10^{03}$	2.4382 $\times \;10^{03}$	3.3497 $\times \;10^{03}$	**2.3443 $\times \;10^{03}$**
F24	3.1860 $\times \;10^{03}$	3.1593 $\times \;10^{03}$	3.1862 $\times \;10^{03}$	3.1353 $\times \;10^{03}$	3.0027 $\times \;10^{03}$	3.1231 $\times \;10^{03}$	3.0056 $\times \;10^{03}$	**2.9823 $\times \;10^{03}$**	2.9992 $\times \;10^{03}$	2.9843 $\times \;10^{03}$
F27	3.3383 $\times \;10^{03}$	3.3188 $\times \;10^{03}$	3.3405 $\times \;10^{03}$	3.3370 $\times \;10^{03}$	3.2671 $\times \;10^{03}$	3.3342 $\times \;10^{03}$	3.2695 $\times \;10^{03}$	3.2749 $\times \;10^{03}$	3.2706 $\times \;10^{03}$	**3.2667 $\times \;10^{03}$**
Average rank	9.58	8.08	9.17	6.08	4.42	5.67	3.25	3.17	3.75	**1.83**
Final rank	10	8	9	7	5	6	3	2	4	**1**

Note: The best results are highlighted in bold.

**Table 3:** CEC2017 test results (Dim = 50).

Function	Metric	HOA	DE	DBO	WOA	PSO	QHDBO	MIDBO
F1	mean	8.5501 $\times \;10^{10}$	1.0703 $\times \;10^{10}$	8.5495 $\times \;10^{08}$	7.9417 $\times \;10^{09}$	1.6788 $\times \;10^{10}$	2.8328 $\times \;10^{08}$	**7.5694 $\times \;10^{07}$**
	std	1.1503 $\times \;10^{10}$	1.2858 $\times \;10^{10}$	5.4221 $\times \;10^{08}$	2.5000 $\times \;10^{09}$	3.1106 $\times \;10^{09}$	3.8752 $\times \;10^{08}$	**1.6702 $\times \;10^{08}$**
	rank	7	5	3	4	6	2	**1**
F3	mean	1.5060 $\times \;10^{05}$	5.4257 $\times \;10^{05}$	2.0660 $\times \;10^{05}$	2.4727 $\times \;10^{05}$	1.6887 $\times \;10^{05}$	2.7622 $\times \;10^{05}$	**1.3188 $\times \;10^{05}$**
	std	**1.6112 $\times \;10^{04}$**	1.6836 $\times \;10^{05}$	3.7777 $\times \;10^{04}$	7.9227 $\times \;10^{04}$	3.7158 $\times \;10^{04}$	6.2939 $\times \;10^{04}$	2.2485 $\times \;10^{04}$
	rank	2	7	4	5	3	6	**1**
F4	mean	2.1229 $\times \;10^{04}$	2.2476 $\times \;10^{03}$	9.3666 $\times \;10^{02}$	2.4055 $\times \;10^{03}$	1.8142 $\times \;10^{03}$	7.4855 $\times \;10^{02}$	**6.6259 $\times \;10^{02}$**
	std	4.3562 $\times \;10^{03}$	2.0552 $\times \;10^{03}$	2.3795 $\times \;10^{02}$	5.5134 $\times \;10^{02}$	6.6578 $\times \;10^{02}$	1.0029 $\times \;10^{02}$	**6.8947 $\times \;10^{01}$**
	rank	7	4	3	6	5	2	**1**
F5	mean	1.0831 $\times \;10^{03}$	8.8866 $\times \;10^{02}$	9.5324 $\times \;10^{02}$	1.0997 $\times \;10^{03}$	9.7246 $\times \;10^{02}$	8.6202 $\times \;10^{02}$	**8.2331 $\times \;10^{02}$**
	std	3.6603 $\times \;10^{01}$	8.6824 $\times \;10^{01}$	1.0332 $\times \;10^{02}$	7.6843 $\times \;10^{01}$	**2.6504 $\times \;10^{01}$**	5.5533 $\times \;10^{01}$	5.0245 $\times \;10^{01}$
	rank	6	3	4	7	5	2	**1**
F6	mean	6.8011 $\times \;10^{02}$	6.4660 $\times \;10^{02}$	6.6350 $\times \;10^{02}$	6.9150 $\times \;10^{02}$	**6.4470 $\times \;10^{02}$**	6.5259 $\times \;10^{02}$	6.4980 $\times \;10^{02}$
	std	7.0286 $\times \;10^{00}$	1.3926 $\times \;10^{01}$	1.0441 $\times \;10^{01}$	1.0893 $\times \;10^{01}$	9.4866 $\times \;10^{00}$	8.8893 $\times \;10^{00}$	**7.0185 $\times \;10^{00}$**
	rank	6	2	5	7	**1**	4	3
F7	mean	1.8284 $\times \;10^{03}$	2.0185 $\times \;10^{03}$	1.4199 $\times \;10^{03}$	1.8787 $\times \;10^{03}$	1.5635 $\times \;10^{03}$	**1.3630 $\times \;10^{03}$**	1.4381 $\times \;10^{03}$
	std	6.9015 $\times \;10^{01}$	3.5056 $\times \;10^{02}$	2.0400 $\times \;10^{02}$	1.1413 $\times \;10^{02}$	**5.0765 $\times \;10^{01}$**	1.0252 $\times \;10^{02}$	1.3456 $\times \;10^{02}$
	rank	5	6	3	7	4	**1**	2
F8	mean	1.4127 $\times \;10^{03}$	1.2143 $\times \;10^{03}$	1.2947 $\times \;10^{03}$	1.3955 $\times \;10^{03}$	1.2794 $\times \;10^{03}$	1.1445 $\times \;10^{03}$	**1.1120 $\times \;10^{03}$**
	std	4.4990 $\times \;10^{01}$	1.0119 $\times \;10^{02}$	9.0161 $\times \;10^{01}$	8.5121 $\times \;10^{01}$	**2.7752 $\times \;10^{01}$**	4.1225 $\times \;10^{01}$	4.2551 $\times \;10^{01}$
	rank	7	3	5	6	4	2	**1**
F9	mean	2.5961 $\times \;10^{04}$	2.5891 $\times \;10^{04}$	2.2432 $\times \;10^{04}$	3.4267 $\times \;10^{04}$	**1.2187 $\times \;10^{04}$**	2.1279 $\times \;10^{04}$	1.2867 $\times \;10^{04}$
	std	**4.5413 $\times \;10^{03}$**	9.5442 $\times \;10^{03}$	6.9474 $\times \;10^{03}$	7.7342 $\times \;10^{03}$	5.4466 $\times \;10^{03}$	1.1476 $\times \;10^{04}$	5.4010 $\times \;10^{03}$
	rank	5	6	4	7	**1**	3	2
F10	mean	1.3227 $\times \;10^{04}$	1.3139 $\times \;10^{04}$	1.0490 $\times \;10^{04}$	1.2749 $\times \;10^{04}$	1.4697 $\times \;10^{04}$	1.0735 $\times \;10^{04}$	**9.4563 $\times \;10^{03}$**
	std	8.7954 $\times \;10^{02}$	1.5325 $\times \;10^{03}$	2.3260 $\times \;10^{03}$	1.2102 $\times \;10^{03}$	**4.8484 $\times \;10^{02}$**	2.9806 $\times \;10^{03}$	1.5827 $\times \;10^{03}$
	rank	6	5	2	4	7	3	**1**
F11	mean	1.8849 $\times \;10^{04}$	3.2673 $\times \;10^{04}$	2.8221 $\times \;10^{03}$	5.3163 $\times \;10^{03}$	3.9096 $\times \;10^{03}$	1.8923 $\times \;10^{03}$	**1.5796 $\times \;10^{03}$**
	std	2.4992 $\times \;10^{03}$	2.5130 $\times \;10^{04}$	1.8224 $\times \;10^{03}$	1.4180 $\times \;10^{03}$	7.4525 $\times \;10^{02}$	**3.0039 $\times \;10^{02}$**	3.5460 $\times \;10^{02}$
	rank	6	7	3	5	4	2	**1**
F12	mean	4.7782 $\times \;10^{10}$	1.4146 $\times \;10^{09}$	3.8830 $\times \;10^{08}$	1.8606 $\times \;10^{09}$	5.8107 $\times \;10^{09}$	1.9902 $\times \;10^{08}$	**4.0991 $\times \;10^{07}$**
	std	9.1222 $\times \;10^{09}$	2.3082 $\times \;10^{09}$	4.8494 $\times \;10^{08}$	6.3968 $\times \;10^{08}$	2.7816 $\times \;10^{09}$	1.6713 $\times \;10^{08}$	**3.8590 $\times \;10^{07}$**
	rank	7	4	3	5	6	2	**1**
F13	mean	2.4779 $\times \;10^{10}$	6.4374 $\times \;10^{08}$	3.1567 $\times \;10^{07}$	1.1138 $\times \;10^{08}$	1.4752 $\times \;10^{09}$	5.5005 $\times \;10^{06}$	**1.2803 $\times \;10^{05}$**
	std	8.9960 $\times \;10^{09}$	1.4725 $\times \;10^{09}$	4.5117 $\times \;10^{07}$	7.7859 $\times \;10^{07}$	1.2828 $\times \;10^{09}$	7.7124 $\times \;10^{06}$	**1.1488 $\times \;10^{05}$**
	rank	7	3	4	5	6	2	**1**
F14	mean	5.6274 $\times \;10^{07}$	6.2251 $\times \;10^{06}$	3.3208 $\times \;10^{06}$	4.5881 $\times \;10^{06}$	2.2088 $\times \;10^{06}$	1.8594 $\times \;10^{06}$	**1.1050 $\times \;10^{06}$**
	std	3.8611 $\times \;10^{07}$	1.2230 $\times \;10^{07}$	3.3273 $\times \;10^{06}$	4.3184 $\times \;10^{06}$	4.0268 $\times \;10^{06}$	1.6278 $\times \;10^{06}$	**6.9628 $\times \;10^{05}$**
	rank	7	2	5	6	4	3	**1**
F15	mean	4.1982 $\times \;10^{09}$	4.0295 $\times \;10^{07}$	4.4859 $\times \;10^{07}$	1.7444 $\times \;10^{07}$	3.1713 $\times \;10^{08}$	1.2247 $\times \;10^{05}$	**4.1742 $\times \;10^{04}$**
	std	2.7023 $\times \;10^{09}$	1.9422 $\times \;10^{08}$	1.4271 $\times \;10^{08}$	2.2923 $\times \;10^{07}$	7.2589 $\times \;10^{07}$	6.9388 $\times \;10^{04}$	**2.6410 $\times \;10^{04}$**
	rank	7	2	4	5	6	3	**1**
F16	mean	7.1270 $\times \;10^{03}$	4.5082 $\times \;10^{03}$	4.6632 $\times \;10^{03}$	5.9137 $\times \;10^{03}$	5.0266 $\times \;10^{03}$	4.7454 $\times \;10^{03}$	**3.8350 $\times \;10^{03}$**
	std	8.9921 $\times \;10^{02}$	7.2752 $\times \;10^{02}$	6.8366 $\times \;10^{02}$	9.7806 $\times \;10^{02}$	**3.8689 $\times \;10^{02}$**	7.4447 $\times \;10^{02}$	4.7648 $\times \;10^{02}$
	rank	7	2	3	6	5	4	**1**
F17	mean	4.9343 $\times \;10^{03}$	4.2452 $\times \;10^{03}$	4.2215 $\times \;10^{03}$	4.3360 $\times \;10^{03}$	4.4604 $\times \;10^{03}$	3.8339 $\times \;10^{03}$	**3.4381 $\times \;10^{03}$**
	std	7.5421 $\times \;10^{02}$	7.1008 $\times \;10^{02}$	5.3508 $\times \;10^{02}$	5.6636 $\times \;10^{02}$	2.5988 $\times \;10^{02}$	4.4537 $\times \;10^{02}$	**2.3454 $\times \;10^{02}$**
	rank	7	3	4	5	6	2	**1**
F18	mean	1.0312 $\times \;10^{08}$	4.5927 $\times \;10^{07}$	7.9394 $\times \;10^{06}$	3.8041 $\times \;10^{07}$	1.2055 $\times \;10^{07}$	6.7218 $\times \;10^{06}$	**4.3365 $\times \;10^{06}$**
	std	5.1922 $\times \;10^{07}$	7.2484 $\times \;10^{07}$	1.0713 $\times \;10^{07}$	3.2022 $\times \;10^{07}$	4.5469 $\times \;10^{06}$	1.2256 $\times \;10^{07}$	**3.2555 $\times \;10^{06}$**
	rank	7	5	3	6	4	2	**1**
F19	mean	1.1333 $\times \;10^{09}$	1.3032 $\times \;10^{07}$	6.8058 $\times \;10^{06}$	7.1569 $\times \;10^{06}$	1.6059 $\times \;10^{08}$	1.3002 $\times \;10^{06}$	**7.7778 $\times \;10^{04}$**
	std	8.9663 $\times \;10^{08}$	3.8916 $\times \;10^{07}$	9.6921 $\times \;10^{06}$	7.2814 $\times \;10^{06}$	4.5921 $\times \;10^{07}$	1.6547 $\times \;10^{06}$	**1.1138 $\times \;10^{05}$**
	rank	7	2	4	5	6	3	**1**
F20	mean	3.6182 $\times \;10^{03}$	4.4082 $\times \;10^{03}$	3.8125 $\times \;10^{03}$	3.9643 $\times \;10^{03}$	3.9237 $\times \;10^{03}$	3.7196 $\times \;10^{03}$	**3.2433 $\times \;10^{03}$**
	std	2.7673 $\times \;10^{02}$	5.6270 $\times \;10^{02}$	3.1105 $\times \;10^{02}$	2.5650 $\times \;10^{02}$	**2.2477 $\times \;10^{02}$**	4.2420 $\times \;10^{02}$	3.8337 $\times \;10^{02}$
	rank	2	7	4	5	6	3	**1**
F21	mean	2.9785 $\times \;10^{03}$	2.6972 $\times \;10^{03}$	2.8510 $\times \;10^{03}$	2.9934 $\times \;10^{03}$	2.7927 $\times \;10^{03}$	2.8806 $\times \;10^{03}$	**2.6001 $\times \;10^{03}$**
	std	7.3108 $\times \;10^{01}$	9.3352 $\times \;10^{01}$	1.0139 $\times \;10^{02}$	8.4173 $\times \;10^{01}$	**3.9788 $\times \;10^{01}$**	1.1817 $\times \;10^{02}$	6.3260 $\times \;10^{01}$
	rank	7	2	4	6	3	5	**1**
F22	mean	1.4980 $\times \;10^{04}$	1.5366 $\times \;10^{04}$	1.1557 $\times \;10^{04}$	1.4498 $\times \;10^{04}$	1.6500 $\times \;10^{04}$	1.2125 $\times \;10^{04}$	**1.0351 $\times \;10^{04}$**
	std	**1.0407 $\times \;10^{03}$**	1.8469 $\times \;10^{03}$	2.6051 $\times \;10^{03}$	1.1381 $\times \;10^{03}$	4.9895 $\times \;10^{02}$	2.0855 $\times \;10^{03}$	3.2066 $\times \;10^{03}$
	rank	5	6	2	4	7	3	**1**
F23	mean	4.5101 $\times \;10^{03}$	3.2081 $\times \;10^{03}$	3.4950 $\times \;10^{03}$	3.8239 $\times \;10^{03}$	3.4571 $\times \;10^{03}$	3.4869 $\times \;10^{03}$	**3.1820 $\times \;10^{03}$**
	std	2.1190 $\times \;10^{02}$	9.7704 $\times \;10^{01}$	1.4288 $\times \;10^{02}$	1.7085 $\times \;10^{02}$	1.1261 $\times \;10^{02}$	2.2048 $\times \;10^{02}$	**9.1383 $\times \;10^{01}$**
	rank	7	2	5	6	4	3	**1**
F24	mean	5.0335 $\times \;10^{03}$	3.3022 $\times \;10^{03}$	3.6881 $\times \;10^{03}$	3.8560 $\times \;10^{03}$	3.6574 $\times \;10^{03}$	3.5858 $\times \;10^{03}$	**3.2918 $\times \;10^{03}$**
	std	2.6841 $\times \;10^{02}$	1.0355 $\times \;10^{02}$	1.7572 $\times \;10^{02}$	1.4696 $\times \;10^{02}$	1.6632 $\times \;10^{02}$	1.7405 $\times \;10^{02}$	**8.1293 $\times \;10^{01}$**
	rank	7	2	5	6	4	3	**1**
F25	mean	1.1711 $\times \;10^{04}$	4.3564 $\times \;10^{03}$	3.2447 $\times \;10^{03}$	4.1799 $\times \;10^{03}$	3.8277 $\times \;10^{03}$	3.1547 $\times \;10^{03}$	**3.1516 $\times \;10^{03}$**
	std	1.0519 $\times \;10^{03}$	1.5789 $\times \;10^{03}$	1.5819 $\times \;10^{02}$	3.6394 $\times \;10^{02}$	2.2231 $\times \;10^{02}$	4.9728 $\times \;10^{01}$	**3.8338 $\times \;10^{01}$**
	rank	7	5	3	6	4	2	**1**
F26	mean	1.5821 $\times \;10^{04}$	8.9820 $\times \;10^{03}$	9.1528 $\times \;10^{03}$	1.4149 $\times \;10^{04}$	8.3066 $\times \;10^{03}$	7.9520 $\times \;10^{03}$	**7.5113 $\times \;10^{03}$**
	std	**6.4252 $\times \;10^{02}$**	1.2961 $\times \;10^{03}$	1.9386 $\times \;10^{03}$	1.4740 $\times \;10^{03}$	1.5008 $\times \;10^{03}$	2.6612 $\times \;10^{03}$	2.5289 $\times \;10^{03}$
	rank	7	4	5	6	3	2	**1**
F27	mean	7.0429 $\times \;10^{03}$	**3.6814 $\times \;10^{03}$**	4.1240 $\times \;10^{03}$	4.6575 $\times \;10^{03}$	3.7278 $\times \;10^{03}$	3.9018 $\times \;10^{03}$	3.6976 $\times \;10^{03}$
	std	5.8660 $\times \;10^{02}$	1.4029 $\times \;10^{02}$	3.1507 $\times \;10^{02}$	6.3587 $\times \;10^{02}$	2.2514 $\times \;10^{02}$	2.4758 $\times \;10^{02}$	**1.3074 $\times \;10^{02}$**
	rank	7	2	5	6	3	4	**1**
F28	mean	1.0616 $\times \;10^{04}$	6.3288 $\times \;10^{03}$	5.8985 $\times \;10^{03}$	5.1477 $\times \;10^{03}$	4.5125 $\times \;10^{03}$	3.8613 $\times \;10^{03}$	**3.4346 $\times \;10^{03}$**
	std	7.9570 $\times \;10^{02}$	1.7491 $\times \;10^{03}$	2.0751 $\times \;10^{03}$	3.3643 $\times \;10^{02}$	8.9637 $\times \;10^{02}$	1.1724 $\times \;10^{03}$	**5.9516 $\times \;10^{01}$**
	rank	7	6	4	5	3	2	**1**
F29	mean	1.8839 $\times \;10^{04}$	5.5454 $\times \;10^{03}$	6.2820 $\times \;10^{03}$	8.3695 $\times \;10^{03}$	6.2301 $\times \;10^{03}$	5.7750 $\times \;10^{03}$	**5.2988 $\times \;10^{03}$**
	std	1.1022 $\times \;10^{04}$	6.8123 $\times \;10^{02}$	9.0411 $\times \;10^{02}$	1.2495 $\times \;10^{03}$	**3.2188 $\times \;10^{02}$**	6.5789 $\times \;10^{02}$	4.1837 $\times \;10^{02}$
	rank	7	2	5	6	4	3	**1**
F30	mean	3.0313 $\times \;10^{09}$	2.9087 $\times \;10^{08}$	4.0330 $\times \;10^{07}$	2.6642 $\times \;10^{08}$	4.4150 $\times \;10^{08}$	1.6624 $\times \;10^{07}$	**3.3476 $\times \;10^{06}$**
	std	1.5907 $\times \;10^{09}$	1.0033 $\times \;10^{09}$	3.3621 $\times \;10^{07}$	9.3361 $\times \;10^{07}$	8.8401 $\times \;10^{07}$	1.5359 $\times \;10^{07}$	**2.5463 $\times \;10^{06}$**
	rank	7	4	3	5	6	2	**1**
Average rank		6.3103	3.8966	3.8276	5.8621	4.4828	2.7586	**1.1379**
Final rank		7	4	3	6	5	2	1

Note: The best results for each metric are shown in bold.

**Table 4:** Wilcoxon rank sum test.

Function	MIDBO vs. HOA	MIDBO vs. DE	MIDBO vs. DBO	MIDBO vs. WOA	MIDBO vs. PSO	MIDBO vs. QHDBO
F1	3.0199 $\times \;10^{-11}$	3.0199 $\times \;10^{-11}$	1.4110 $\times \;10^{-09}$	3.0199 $\times \;10^{-11}$	3.0199 $\times \;10^{-11}$	2.6784 $\times \;10^{-06}$
F3	5.8737 $\times \;10^{-04}$	3.0199 $\times \;10^{-11}$	4.1997 $\times \;10^{-10}$	6.1210 $\times \;10^{-10}$	1.2493 $\times \;10^{-05}$	3.0199 $\times \;10^{-11}$
F4	3.0199 $\times \;10^{-11}$	1.6132 $\times \;10^{-10}$	2.3897 $\times \;10^{-08}$	3.0199 $\times \;10^{-11}$	3.0199 $\times \;10^{-11}$	3.1821 $\times \;10^{-04}$
F5	3.0199 $\times \;10^{-11}$	1.1143 $\times \;10^{-03}$	1.8608 $\times \;10^{-06}$	3.0199 $\times \;10^{-11}$	3.0199 $\times \;10^{-11}$	5.3221 $\times \;10^{-03}$
F6	3.0199 $\times \;10^{-11}$	**5.5546 $\times \;10^{-02}$**	2.1540 $\times \;10^{-06}$	3.0199 $\times \;10^{-11}$	1.5969 $\times \;10^{-03}$	**3.4783 $\times \;10^{-01}$**
F7	3.0199 $\times \;10^{-11}$	2.2273 $\times \;10^{-09}$	**5.9969 $\times \;10^{-01}$**	3.6897 $\times \;10^{-11}$	1.0907 $\times \;10^{-05}$	1.1711 $\times \;10^{-02}$
F8	3.0199 $\times \;10^{-11}$	2.5974 $\times \;10^{-05}$	1.0702 $\times \;10^{-09}$	3.0199 $\times \;10^{-11}$	3.0199 $\times \;10^{-11}$	3.3386 $\times \;10^{-03}$
F9	2.9215 $\times \;10^{-09}$	1.0666 $\times \;10^{-07}$	1.4918 $\times \;10^{-06}$	1.0937 $\times \;10^{-10}$	**4.4642 $\times \;10^{-01}$**	3.8481 $\times \;10^{-03}$
F10	1.7769 $\times \;10^{-10}$	2.9215 $\times \;10^{-09}$	**1.0869 $\times \;10^{-01}$**	2.6695 $\times \;10^{-09}$	3.0199 $\times \;10^{-11}$	**2.2823 $\times \;10^{-01}$**
F11	3.0199 $\times \;10^{-11}$	3.3384 $\times \;10^{-11}$	1.5581 $\times \;10^{-08}$	3.0199 $\times \;10^{-11}$	4.0772 $\times \;10^{-11}$	7.7387 $\times \;10^{-06}$
F12	3.0199 $\times \;10^{-11}$	1.2870 $\times \;10^{-09}$	8.4948 $\times \;10^{-09}$	3.0199 $\times \;10^{-11}$	3.0199 $\times \;10^{-11}$	4.8011 $\times \;10^{-07}$
F13	3.0199 $\times \;10^{-11}$	4.3531 $\times \;10^{-05}$	8.8910 $\times \;10^{-10}$	3.0199 $\times \;10^{-11}$	3.0199 $\times \;10^{-11}$	8.8910 $\times \;10^{-10}$
F14	3.0199 $\times \;10^{-11}$	**3.3285 $\times \;10^{-01}$**	1.6798 $\times \;10^{-03}$	7.0881 $\times \;10^{-08}$	2.8129 $\times \;10^{-02}$	**6.1452 $\times \;10^{-02}$**
F15	3.0199 $\times \;10^{-11}$	**2.1702 $\times \;10^{-01}$**	2.1947 $\times \;10^{-08}$	3.0199 $\times \;10^{-11}$	3.0199 $\times \;10^{-11}$	2.1959 $\times \;10^{-07}$
F16	3.0199 $\times \;10^{-11}$	5.2640 $\times \;10^{-04}$	3.3242 $\times \;10^{-06}$	1.7769 $\times \;10^{-10}$	3.1589 $\times \;10^{-10}$	1.8608 $\times \;10^{-06}$
F17	2.6099 $\times \;10^{-10}$	8.8411 $\times \;10^{-07}$	1.7294 $\times \;10^{-07}$	2.0152 $\times \;10^{-08}$	1.9568 $\times \;10^{-10}$	4.7138 $\times \;10^{-04}$
F18	3.0199 $\times \;10^{-11}$	1.6062 $\times \;10^{-06}$	**5.5923 $\times \;10^{-01}$**	1.1737 $\times \;10^{-09}$	2.3897 $\times \;10^{-08}$	**2.5188 $\times \;10^{-01}$**
F19	3.0199 $\times \;10^{-11}$	8.3146 $\times \;10^{-03}$	1.0105 $\times \;10^{-08}$	3.0199 $\times \;10^{-11}$	3.0199 $\times \;10^{-11}$	2.2273 $\times \;10^{-09}$
F20	9.2113 $\times \;10^{-05}$	1.2870 $\times \;10^{-09}$	4.8011 $\times \;10^{-07}$	3.0199 $\times \;10^{-11}$	1.5581 $\times \;10^{-08}$	9.2113 $\times \;10^{-05}$
F21	3.0199 $\times \;10^{-11}$	2.9590 $\times \;10^{-05}$	8.1527 $\times \;10^{-11}$	3.0199 $\times \;10^{-11}$	8.9934 $\times \;10^{-11}$	1.0937 $\times \;10^{-10}$
F22	5.9673 $\times \;10^{-09}$	4.5726 $\times \;10^{-09}$	**1.1199 $\times \;10^{-01}$**	2.8314 $\times \;10^{-08}$	9.9186 $\times \;10^{-11}$	1.6285 $\times \;10^{-02}$
F23	3.0199 $\times \;10^{-11}$	**2.6433 $\times \;10^{-01}$**	6.5183 $\times \;10^{-09}$	3.3384 $\times \;10^{-11}$	1.2023 $\times \;10^{-08}$	1.3594 $\times \;10^{-07}$
F24	3.0199 $\times \;10^{-11}$	**9.5873 $\times \;10^{-01}$**	1.2057 $\times \;10^{-10}$	3.0199 $\times \;10^{-11}$	3.6897 $\times \;10^{-11}$	1.0702 $\times \;10^{-09}$
F25	3.0199 $\times \;10^{-11}$	3.6897 $\times \;10^{-11}$	1.7666 $\times \;10^{-03}$	3.0199 $\times \;10^{-11}$	3.0199 $\times \;10^{-11}$	**1.0000 $\times \;10^{00}$**
F26	3.0199 $\times \;10^{-11}$	**6.1452 $\times \;10^{-02}$**	1.5638 $\times \;10^{-02}$	3.6897 $\times \;10^{-11}$	**3.2553 $\times \;10^{-01}$**	**4.4642 $\times \;10^{-01}$**
F27	3.0199 $\times \;10^{-11}$	**6.9522 $\times \;10^{-01}$**	9.0632 $\times \;10^{-08}$	8.1527 $\times \;10^{-11}$	**7.2827 $\times \;10^{-01}$**	2.8389 $\times \;10^{-04}$
F28	3.0199 $\times \;10^{-11}$	1.2057 $\times \;10^{-10}$	9.9186 $\times \;10^{-11}$	3.0199 $\times \;10^{-11}$	3.0199 $\times \;10^{-11}$	1.2493 $\times \;10^{-05}$
F29	3.0199 $\times \;10^{-11}$	**1.7613 $\times \;10^{-01}$**	1.0188 $\times \;10^{-05}$	3.3384 $\times \;10^{-11}$	6.7220 $\times \;10^{-10}$	1.0315 $\times \;10^{-02}$
F30	3.0199 $\times \;10^{-11}$	2.1947 $\times \;10^{-08}$	8.1527 $\times \;10^{-11}$	3.0199 $\times \;10^{-11}$	3.0199 $\times \;10^{-11}$	1.4294 $\times \;10^{-08}$
+/=/–	29 / 0 / 0	21 / 8 / 0	25 / 4 / 0	29 / 0 / 0	25 / 3 / 1	22 / 6 / 1

**Table 5:** CEC2022 test results (Dim = 10).

Function	Metric	HOA	DE	DBO	WOA	PSO	QHDBO	MIDBO
F1	mean	1.7064 $\times \;10^{04}$	3.2804 $\times \;10^{04}$	1.5248 $\times \;10^{03}$	1.6810 $\times \;10^{04}$	1.0413 $\times \;10^{03}$	1.9943 $\times \;10^{03}$	**3.0123 $\times \;10^{02}$**
	std	5.6621 $\times \;10^{03}$	2.1361 $\times \;10^{04}$	1.0832 $\times \;10^{03}$	1.4218 $\times \;10^{04}$	4.5302 $\times \;10^{02}$	1.8545 $\times \;10^{03}$	**1.4402 $\times \;10^{00}$**
	rank	6	7	4	5	2	3	**1**
F2	mean	9.2853 $\times \;10^{02}$	4.2406 $\times \;10^{02}$	4.2942 $\times \;10^{02}$	4.5524 $\times \;10^{02}$	4.2920 $\times \;10^{02}$	4.1376 $\times \;10^{02}$	**4.0688 $\times \;10^{02}$**
	std	3.1368 $\times \;10^{02}$	6.0127 $\times \;10^{01}$	4.6766 $\times \;10^{01}$	5.5145 $\times \;10^{01}$	3.9029 $\times \;10^{01}$	2.6473 $\times \;10^{01}$	**1.4363 $\times \;10^{01}$**
	rank	7	3	4	5	6	2	**1**
F3	mean	6.2104 $\times \;10^{02}$	6.0802 $\times \;10^{02}$	**6.0512 $\times \;10^{02}$**	6.2729 $\times \;10^{02}$	6.1053 $\times \;10^{02}$	6.0563 $\times \;10^{02}$	6.0570 $\times \;10^{02}$
	std	5.1745 $\times \;10^{00}$	1.0092 $\times \;10^{01}$	5.7223 $\times \;10^{00}$	1.2314 $\times \;10^{01}$	**2.3142 $\times \;10^{00}$**	5.0129 $\times \;10^{00}$	3.9824 $\times \;10^{00}$
	rank	6	4	**1**	7	5	2	3
F4	mean	8.4058 $\times \;10^{02}$	8.3120 $\times \;10^{02}$	8.3168 $\times \;10^{02}$	8.4939 $\times \;10^{02}$	8.3382 $\times \;10^{02}$	8.3740 $\times \;10^{02}$	**8.2601 $\times \;10^{02}$**
	std	7.1790 $\times \;10^{00}$	1.1006 $\times \;10^{01}$	1.2597 $\times \;10^{01}$	1.7514 $\times \;10^{01}$	**5.6920 $\times \;10^{00}$**	1.4188 $\times \;10^{01}$	8.5509 $\times \;10^{00}$
	rank	6	3	2	7	4	5	**1**
F5	mean	2.0645 $\times \;10^{03}$	1.1928 $\times \;10^{03}$	1.1292 $\times \;10^{03}$	2.2127 $\times \;10^{03}$	**9.7276 $\times \;10^{02}$**	1.2190 $\times \;10^{03}$	1.0115 $\times \;10^{03}$
	std	6.3730 $\times \;10^{02}$	2.8676 $\times \;10^{02}$	2.6854 $\times \;10^{02}$	8.6516 $\times \;10^{02}$	**3.1363 $\times \;10^{01}$**	3.9367 $\times \;10^{02}$	1.4081 $\times \;10^{02}$
	rank	6	5	3	7	2	4	**1**
F6	mean	4.2760 $\times \;10^{05}$	6.4884 $\times \;10^{04}$	8.6806 $\times \;10^{03}$	5.4586 $\times \;10^{03}$	6.3540 $\times \;10^{05}$	8.7718 $\times \;10^{03}$	**3.8056 $\times \;10^{03}$**
	std	7.9737 $\times \;10^{05}$	2.0806 $\times \;10^{05}$	5.0788 $\times \;10^{03}$	4.1045 $\times \;10^{03}$	4.8372 $\times \;10^{05}$	5.4267 $\times \;10^{03}$	**3.2408 $\times \;10^{03}$**
	rank	6	5	4	2	7	3	**1**
F7	mean	2.0745 $\times \;10^{03}$	2.0705 $\times \;10^{03}$	2.0441 $\times \;10^{03}$	2.0603 $\times \;10^{03}$	2.0534 $\times \;10^{03}$	2.0511 $\times \;10^{03}$	**2.0372 $\times \;10^{03}$**
	std	3.8530 $\times \;10^{01}$	3.8261 $\times \;10^{01}$	1.5445 $\times \;10^{01}$	2.0246 $\times \;10^{01}$	3.0430 $\times \;10^{01}$	1.9888 $\times \;10^{01}$	**1.1743 $\times \;10^{01}$**
	rank	7	6	2	5	3	4	**1**
F8	mean	2.2357 $\times \;10^{03}$	2.2441 $\times \;10^{03}$	2.2309 $\times \;10^{03}$	2.2446 $\times \;10^{03}$	2.2298 $\times \;10^{03}$	2.2295 $\times \;10^{03}$	**2.2271 $\times \;10^{03}$**
	std	1.5262 $\times \;10^{01}$	3.5131 $\times \;10^{01}$	1.0339 $\times \;10^{01}$	2.7851 $\times \;10^{01}$	**2.9796 $\times \;10^{00}$**	8.4267 $\times \;10^{00}$	7.6904 $\times \;10^{00}$
	rank	4	6	3	7	5	2	**1**
F9	mean	2.4420 $\times \;10^{03}$	2.4485 $\times \;10^{03}$	**2.4000 $\times \;10^{03}$**	2.4091 $\times \;10^{03}$	2.5517 $\times \;10^{03}$	**2.4000 $\times \;10^{03}$**	**2.4000 $\times \;10^{03}$**
	std	3.3310 $\times \;10^{01}$	6.1344 $\times \;10^{01}$	1.0706 $\times \;10^{-09}$	3.6253 $\times \;10^{00}$	5.6752 $\times \;10^{-01}$	**8.6941 $\times \;10^{-13}$**	3.9602 $\times \;10^{-04}$
	rank	6	5	2	4	7	**1**	3
F10	mean	2.5136 $\times \;10^{03}$	3.0926 $\times \;10^{03}$	2.5015 $\times \;10^{03}$	2.7174 $\times \;10^{03}$	2.5625 $\times \;10^{03}$	2.5003 $\times \;10^{03}$	**2.5000 $\times \;10^{03}$**
	std	6.6841 $\times \;10^{00}$	3.3752 $\times \;10^{02}$	3.6086 $\times \;10^{00}$	3.8502 $\times \;10^{02}$	1.0694 $\times \;10^{00}$	1.0524 $\times \;10^{00}$	**1.3236 $\times \;10^{-03}$**
	rank	4	7	2	5	6	**1**	3
F11	mean	2.7084 $\times \;10^{03}$	2.6152 $\times \;10^{03}$	2.6001 $\times \;10^{03}$	2.6030 $\times \;10^{03}$	2.6472 $\times \;10^{03}$	2.6001 $\times \;10^{03}$	**2.6000 $\times \;10^{03}$**
	std	4.5881 $\times \;10^{01}$	2.1023 $\times \;10^{01}$	3.3402 $\times \;10^{-01}$	1.2130 $\times \;10^{00}$	4.5366 $\times \;10^{00}$	7.7601 $\times \;10^{-02}$	**1.3510 $\times \;10^{-02}$**
	rank	7	4	3	5	6	2	**1**
F12	mean	2.9588 $\times \;10^{03}$	2.9546 $\times \;10^{03}$	2.9545 $\times \;10^{03}$	2.9547 $\times \;10^{03}$	2.9550 $\times \;10^{03}$	2.9547 $\times \;10^{03}$	**2.9544 $\times \;10^{03}$**
	std	1.3617 $\times \;10^{00}$	2.1264 $\times \;10^{-01}$	1.2114 $\times \;10^{-01}$	8.4741 $\times \;10^{-02}$	2.6819 $\times \;10^{-01}$	**4.6252 $\times \;10^{-13}$**	2.9845 $\times \;10^{-02}$
	rank	7	3	2	4	6	5	**1**
Average rank		6	4.8333	2.6667	5.25	4.9167	2.8333	**1.5**
Final rank		7	4	2	6	5	3	**1**

Note: The best results for each metric are shown in bold.

**Table 6:** Wilcoxon rank sum test results on the CEC2022 test set.

Function	MIDBO vs. HOA	MIDBO vs. DE	MIDBO vs. DBO	MIDBO vs. WOA	MIDBO vs. PSO	MIDBO vs. QHDBO
F1	3.0199 $\times \;10^{-11}$	3.0199 $\times \;10^{-11}$	6.5261 $\times \;10^{-07}$	3.0199 $\times \;10^{-11}$	3.0199 $\times \;10^{-11}$	5.5999 $\times \;10^{-07}$
F2	3.0199 $\times \;10^{-11}$	**5.3685 $\times \;10^{-02}$**	1.5288 $\times \;10^{-05}$	6.5277 $\times \;10^{-08}$	5.0723 $\times \;10^{-10}$	3.3386 $\times \;10^{-03}$
F3	7.3891 $\times \;10^{-11}$	**9.9410 $\times \;10^{-01}$**	**1.8090 $\times \;10^{-01}$**	1.7769 $\times \;10^{-10}$	3.3242 $\times \;10^{-06}$	**4.9178 $\times \;10^{-01}$**
F4	9.8329 $\times \;10^{-08}$	**7.7272 $\times \;10^{-02}$**	**8.2357 $\times \;10^{-02}$**	3.2555 $\times \;10^{-07}$	2.0058 $\times \;10^{-04}$	1.8575 $\times \;10^{-03}$
F5	8.9934 $\times \;10^{-11}$	1.7649 $\times \;10^{-02}$	**9.6263 $\times \;10^{-02}$**	6.6955 $\times \;10^{-11}$	**8.0727 $\times \;10^{-01}$**	1.3272 $\times \;10^{-02}$
F6	7.0430 $\times \;10^{-07}$	2.1959 $\times \;10^{-07}$	5.0912 $\times \;10^{-06}$	**6.5671 $\times \;10^{-02}$**	3.0199 $\times \;10^{-11}$	5.9706 $\times \;10^{-05}$
F7	3.5201 $\times \;10^{-07}$	2.2780 $\times \;10^{-05}$	**1.2235 $\times \;10^{-01}$**	7.0430 $\times \;10^{-07}$	2.5306 $\times \;10^{-04}$	7.9590 $\times \;10^{-03}$
F8	1.6813 $\times \;10^{-04}$	1.4298 $\times \;10^{-05}$	3.3874 $\times \;10^{-02}$	1.1077 $\times \;10^{-06}$	6.7650 $\times \;10^{-05}$	**1.0869 $\times \;10^{-01}$**
F9	3.0161 $\times \;10^{-11}$	5.1763 $\times \;10^{-07}$	1.4495 $\times \;10^{-09}$	3.0161 $\times \;10^{-11}$	3.0161 $\times \;10^{-11}$	2.9703 $\times \;10^{-11}$
F10	3.0161 $\times \;10^{-11}$	3.0161 $\times \;10^{-11}$	**7.3444 $\times \;10^{-02}$**	3.0161 $\times \;10^{-11}$	3.0161 $\times \;10^{-11}$	2.9422 $\times \;10^{-07}$
F11	3.0199 $\times \;10^{-11}$	2.3866 $\times \;10^{-04}$	1.4932 $\times \;10^{-04}$	3.0199 $\times \;10^{-11}$	3.0199 $\times \;10^{-11}$	**1.2235 $\times \;10^{-01}$**
F12	2.6859 $\times \;10^{-11}$	3.0953 $\times \;10^{-02}$	2.1483 $\times \;10^{-02}$	5.5571 $\times \;10^{-10}$	2.6859 $\times \;10^{-11}$	1.0410 $\times \;10^{-12}$
+/=/−	12 / 0 / 0	9 / 3 / 0	6 / 5 / 1	11 / 1 / 0	11 / 1 / 0	8 / 3 / 1

**Table 7:** Best results for tension–compression spring design issues.

Algorithm	Optimum Variables			Best Cost	Ranking
	*d*	*D*	*P*		
HOA	0.05149041	0.35019476	11.75205863	0.01276821	7
DE	0.05185571	0.36074007	11.05699006	0.01266574	3
DBO	0.05	0.31742547	14.02776195	0.01271905	4
WOA	0.05153875	0.35311241	11.50352908	0.01266567	2
PSO	0.05335566	0.39812189	9.229910776	0.01272780	6
QHDBO	0.05	0.31741769	14.03239520	0.01272242	5
MIDBO	0.05162258	0.35512065	11.38321349	**0.01266531**	**1**

Note: The best results are highlighted in bold.

**Table 8:** Statistical results of tension–compression spring design issues.

Algorithm	Best	Mean	Std	Worst
HOA	0.01276821	0.01422220	0.00109043	0.01705629
DE	0.01266574	0.01418542	0.00364543	0.02883747
DBO	0.01271905	0.01519093	0.00243828	0.01800172
WOA	0.01266567	0.01381182	0.00125510	0.01777675
PSO	0.01272780	0.01325253	0.00115248	0.01802708
QHDBO	0.01272242	3972.16977	12226.0318	39721.5549
MIDBO	**0.01266531**	**0.01287473**	**0.00031892**	**0.01385540**

Note: The best results for each metric are shown in bold.

**Table 9:** Best results for pressure vessel design issues.

Algorithm	Optimum Variables				Best Cost	Ranking
	$T_{s}$	$T_{h}$	R	L		
HOA	14.46908487	9.41544744	46.15582410	132.1020384	6497.639768	7
DE	12.32937401	5.95960812	40.31961891	200	5743.028259	4
DBO	11.78387737	6.36965630	40.31961872	200	5743.028207	2
WOA	12.08028516	7.45382003	40.31964720	199.9996529	5914.381203	5
PSO	12.06999923	6.96648212	40.68269309	196.3779765	5944.359944	6
QHDBO	12.20767452	6.45691444	40.31961872	200	5743.028207	2
MIDBO	11.58768820	5.75856973	40.33293551	199.8148146	**5743.021200**	**1**

**Table 10:** Statistical results of pressure vessel design issues.

Algorithm	Best	Mean	Std	Worst
HOA	6497.639768	7151.331985	377.0241357	7710.421479
DE	5743.028259	6245.583864	556.9250329	7911.454283
DBO	5743.028207	6126.716891	441.4547694	7198.824766
WOA	5914.381203	7846.276499	2547.835593	15978.5745
PSO	5944.359944	6383.284761	**355.8878759**	**7184.192856**
QHDBO	5743.028207	6452.409249	490.5638215	7198.824766
MIDBO	**5743.021200**	**6033.448733**	376.1353799	7198.824766

**Table 11:** Best results for speed reducer design issues.

Algorithm	Optimum Variables							Best Cost	Ranking
	b	m	p	$l_{1}$	$l_{2}$	$d_{1}$	$d_{2}$		
HOA	3.5283902	0.7057965	17.0815174	8.0237040	7.9954109	3.3916207	5.3467375	3108.605405	7
DE	8.1048468	0.7	10.0045627	6.9420956	7.7179078	3.3614295	5.2891139	**2513.700952**	**1**
DBO	3.4990378	0.7	17	8.3	7.7152089	3.3525325	5.2866544	3003.569625	6
WOA	3.4989428	0.7	17.0109922	7.3	7.7118498	3.3554858	5.2866520	2998.523131	4
PSO	3.5007469	0.7	17	7.6511181	7.7863316	3.3636023	5.2877010	3003.403769	5
QHDBO	3.4989630	0.7	17	7.3016166	7.7166102	3.3505713	5.2866612	2994.291185	3
MIDBO	3.4990377	0.7	17	7.3	7.7152089	3.3505410	5.2866544	2994.234252	2

**Table 12:** Statistical results for speed reducer design issues.

Algorithm	Best	Mean	Std	Worst
HOA	3108.605405	7117.868848	7620.555658	28686.3642
DE	**2513.700952**	**2679.437592**	75.53042354	**2898.544731**
DBO	3003.569625	3031.828967	16.15150037	3056.000079
WOA	2998.523131	3227.816987	412.0499696	4863.87868
PSO	3003.403769	3036.148094	16.35192864	3057.403942
QHDBO	2994.291185	6659.698848	8941.285751	31812.79325
MIDBO	2994.234252	2994.234331	**0.000319158**	2994.235682

## Data Availability

No dataset was used in this research.

## Abbreviations

The following abbreviations are used in this manuscript:

HOA	Hiking optimization algorithm
WOA	Whale optimization algorithm
QHDBO	Dung beetle optimization algorithm based on quantum computing and multi-strategy
	fusion
DBO	Dung Beetle Optimizer
Std	Standard deviation
MIDBO	Multi-Strategy Improved Dung Beetle Optimizer
TEO	Thermal exchange optimization
BBO	Biogeography-based optimization
ASO	Atom search optimization
DE	Differential evolution
KOA	Keplerian optimization algorithm
SGO	Social group optimization
LCA	League championship algorithm
SCA	Sine cosine algorithm
SPO	Student psychology optimization
BWO	Beluga whale optimization
PSO	Particle swarm optimization
WO	Walrus optimizer
OBL	Opposition-based learning
HO	Hippopotamus optimization algorithm
GGO	Greylag Goose Optimization
NFL	No free lunch
Mean	Mean value
Best	Best value
Worst	Worst value
